# Investigating the Role of Obesity in Prostate Cancer and Identifying Biomarkers for Drug Discovery: Systems Biology and Deep Learning Approaches

**DOI:** 10.3390/molecules27030900

**Published:** 2022-01-28

**Authors:** Shan-Ju Yeh, Yun-Chen Chung, Bor-Sen Chen

**Affiliations:** Laboratory of Automatic Control, Signal Processing and Systems Biology, Department of Electrical Engineering, National Tsing Hua University, Hsinchu 30013, Taiwan; m793281@gmail.com (S.-J.Y.); ms107061540@gapp.nthu.edu.tw (Y.-C.C.)

**Keywords:** prostate cancer (PCa), lean PCa, obese PCa, multiple-molecule drug, carcinogenic mechanism, deep neural network (DNN)-based DTI model, drug design specifications

## Abstract

Prostate cancer (PCa) is the second most frequently diagnosed cancer for men and is viewed as the fifth leading cause of death worldwide. The body mass index (BMI) is taken as a vital criterion to elucidate the association between obesity and PCa. In this study, systematic methods are employed to investigate how obesity influences the noncutaneous malignancies of PCa. By comparing the core signaling pathways of lean and obese patients with PCa, we are able to investigate the relationships between obesity and pathogenic mechanisms and identify significant biomarkers as drug targets for drug discovery. Regarding drug design specifications, we take drug–target interaction, drug regulation ability, and drug toxicity into account. One deep neural network (DNN)-based drug–target interaction (DTI) model is trained in advance for predicting drug candidates based on the identified biomarkers. In terms of the application of the DNN-based DTI model and the consideration of drug design specifications, we suggest two potential multiple-molecule drugs to prevent PCa (covering lean and obese PCa) and obesity-specific PCa, respectively. The proposed multiple-molecule drugs (apigenin, digoxin, and orlistat) not only help to prevent PCa, suppressing malignant metastasis, but also result in lower production of fatty acids and cholesterol, especially for obesity-specific PCa.

## 1. Introduction

Over the past twelve years, the prevalence of obesity in populations has grown by up to 10.3%. This situation has been even more dramatic in the past five years, with growth of up to approximately 20% [1]. Although there are declining mortality rates from prostate cancer (PCa) in high-income countries, the cancer incidence and death rates are increasing due to excess body weight in developing countries [2]. Following the definition of overweight and obesity based on the investigation of the WHO, we find that abnormal or excessive fat accumulation may influence every aspect of human life. As well as increasing the risk of chronic diseases and cardiovascular diseases, obesity is also a decisive factor in causing a large number of cancer categories [3]. Especially for PCa, obesity has an impact on higher cancer incidence, mortality, and poor prognosis. Bearing in mind the association between obesity and PCa, we took the body mass index (BMI) into consideration as a critical criterion for distinguishing the formation of PCa from that of normal prostate cells [4]. Several studies have shown that a higher BMI is associated with a higher risk of lethal PCa, leading to higher mortality rates in the groups of middle-aged and older men [5,6,7]. Therefore, we explored several aspects of PCa, such as multifactorial aberrations of genetic, epigenetic, microenvironment, and biological signaling cascades, and also the obesity factor, for the purpose of preventing the occurrence of PCa.

According to the previous statistics on noncutaneous malignancies in men [8], PCa is the most common heterogeneous disease, and it presents a complex combination of the deregulation of the insulin growth factor (IGF) and genetic, epigenetic, and microenvironmental factors. One biological mechanism corresponding to obesity and PCa involves alterations in sex-hormone secretion and variation of adipokine signaling [9], which facilitate the progression of PCa on the basis of their interactions with IGF [10]. As the relationship between the level of insulin and the concentration of sex hormones has opposite interactive influence [11], we could propose a hypothesis that the development of poorly differentiated PCa is indirectly promoted to aggressive PCa via the driving force of obesity [12]. Moreover, it is reported that hyperinsulinemia, lowering testosterone and dihydrotestosterone levels, demonstrates a vital connection between obesity and the development of poorly differentiated PCa [11]. It is noted that patients suffering from invasive PCa have the characteristics of higher recurrence and poorer survival rate compared with normal PCa. Many studies have shown that the PCa aggressiveness is related to testosterone level [13,14,15,16], which could help in further investigating the mechanisms for the development of benign prostate hyperplasia (BPH) and PCa. In addition, participating in the interference with adjacent tumor cells, adipocytes are also affected by the PCa cells at the same time as stimulating migration [17,18]. From the above speculation, we could obtain a deep insight into several aspects of carcinogenic mechanisms including endocrine status alteration, intra-abdominal pressure ascension, insulin resistance, and adipokine secretion alteration, which favor the realization of the effects of inflammation on causing high-grade PCa [19]. As a result, based on the carcinogenic molecular mechanism analysis, we were able to identify significant biomarkers for systems drug design.

**Figure 1:**
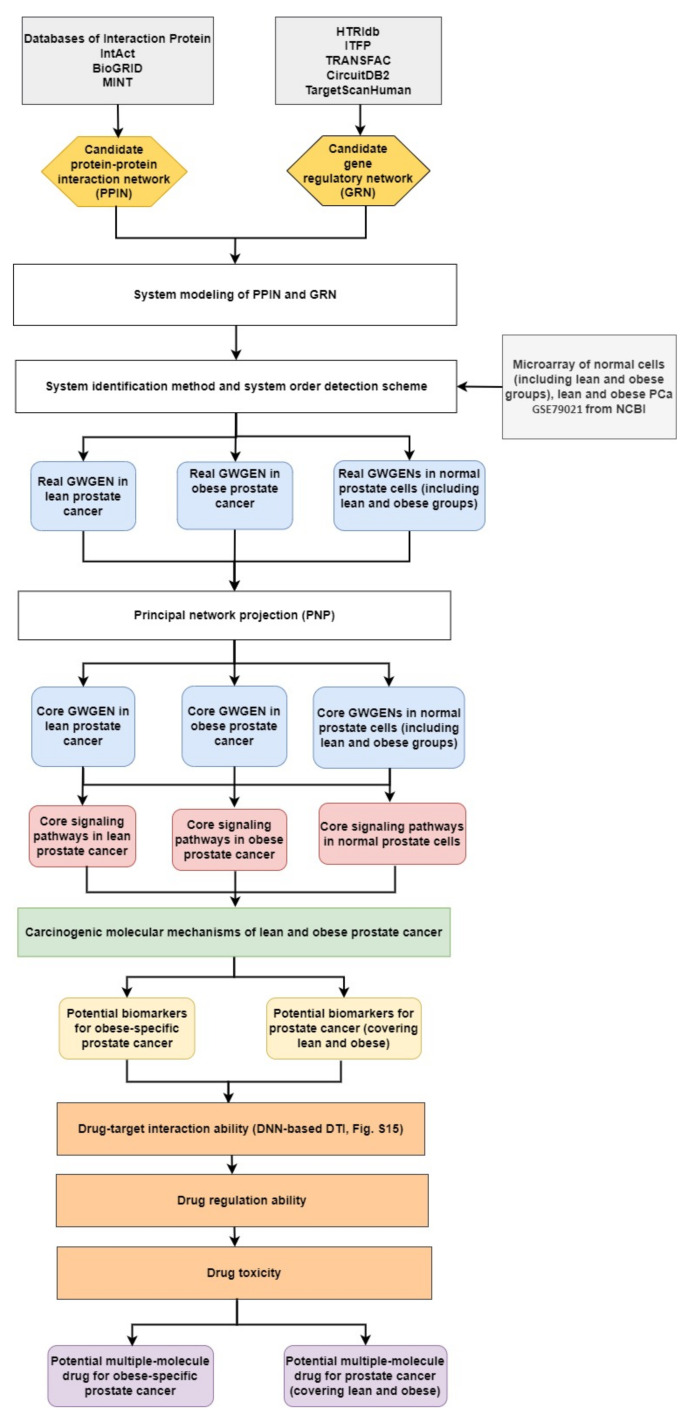
The flowchart for the systems biology approach. The proposed systems biology approach is used to construct the candidate GWGEN, real GWGENs, core GWGENs, and core signaling pathways of two groups of normal (lean and obese) prostate cells, and lean PCa and obese PCa, for finding multiple-molecule drugs targeting identified biomarkers. The yellow hexagonal blocks indicate the candidate protein–protein interaction network (PPIN) constructed by databases DIP, IntAct, BioGRID, and MINT and the candidate gene regulatory network (GRN) built by databases HTRIdb, ITFP, CircuitDB2, TargetScanHuman, and TRANSFAC; the white rectangular blocks indicate the methods of building real GWGENs and extracting the core GWGENs; the grey rectangular block show the databases; the light-blue rounded rectangular blocks are for real GWGENs and core GWGENs in normal prostate cells (including lean and obese groups), and lean and obese PCa, respectively; the red rounded rectangular blocks are core signaling pathways of normal prostate cells (including lean and obese groups), and lean and obese PCa; the yellow rounded rectangular blocks represent potential biomarkers; the orange rectangular blocks denote drug design specifications; the purple blocks are the suggested multiple-molecule drugs for PCa and obesity-specific PCa, respectively.

For analyzing the molecular mechanisms of prostate cancer and identifying essential biomarkers, we needed to fully compare four core genome-wide genetic and epigenetic networks (GWGENs) of normal prostate cells in in the lean group, normal prostate cells in the obese group, lean PCa cells, and obese PCa cells, respectively. Furthermore, a BMI of 25 is considered as the obesity threshold, used for splitting the data into two groups: one for lean people and the other for obese people. The flowchart of the systems biology approach for PCa shown in Figure 1 can be divided into five steps as follows: (1) the construction of a candidate GWGEN; (2) performing system identification and using the system order detection scheme to obtain real GWGENs of normal prostate cells (lean and obese groups), and lean and obese PCa cells, as shown in Figures S1–S4; (3) the extraction of core GWGENs for normal prostate cells (lean and obese groups), and lean and obese PCa cells, as shown in in Figures S5–S8 using the principal network projection (PNP) approach; (4) the comparison of core signaling pathways, as shown in Figures S9–S12, and the investigation of carcinogenic mechanisms for identifying significant biomarkers as drug targets for PCa (covering lean and obese) and obesity-specific PCa; and (5) multiple-molecule drug discovery via drug design specifications including drug–target interaction, drug regulation ability, and drug toxicity toward identified biomarkers. Consequently, we suggested two multiple-molecule drugs, one with apigenin and digoxin for PCa (covering lean and obese) and the other with apigenin, digoxin, and orlistat for obesity-specific PCa.

## 2. Results

### 2.1. Overview of Systems Biology Approaches and Drug Design Specification

**Figure 2:**
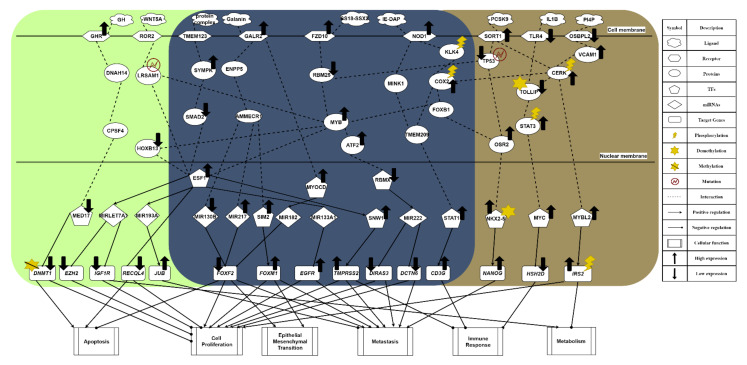
The common and specific core signaling pathways for lean and obese PCa. This figure summarizes the genetic and epigenetic carcinogenic mechanisms of lean and obese PCa. The signaling pathways in the deep blue region are the common core signaling pathways of lean and obese PCa. The light green region represents specific core signaling pathways of lean PCa. The brown region denotes specific core signaling pathways of obese PCa. The black arrow heads of solid lines denote activation of TF, miRNA, target genes, and cellular functions; the black circle heads of solid lines refer to inhibition of TF, miRNA, target genes, and cellular functions; the black up arrows signify high expression of protein, receptor, TF, and target genes; the black down arrows indicate low expression of protein, receptor, TF, and target genes.

For the purpose of understanding the effect of obesity on prostate cancer (PCa) comprehensively, we used a systems biology approach. The related flowchart is shown in Figure 1. Firstly, via big database mining, we constructed a candidate GWGEN. It consisted of a protein–protein interaction network (PPIN) and a gene regulatory network (GRN). We then performed system modeling for proteins, genes, miRNAs, and lncRNAs. In this study, we separated our microarray data into four groups: lean normal, obese normal, lean PCa, and obese PCa. With the help of the microarray data, we performed system identification and used the system order detection scheme to obtain real GWGENs for two groups of normal cells (lean and obese), lean PCa, and obese PCa. The overall statistics for nodes and edges for the candidate GWGEN and the real GWGENs are given in Table S1. Compared to the total number edges in the candidate GWGEN, it is noted that the total number of edges decreased considerably in real GWGENs, since the false positives were removed by the system order detection scheme. However, the real GWGENs (Figures S1–S4) were still too complex to analyze. Hence, the PNP method was used to extract their core GWGENs (Figures S5–S8) by choosing the top 3000 nodes based on their 2-norm projection values in the 85%-significant network energy structure, to narrow down the real GWGENs. In order to investigate carcinogenic mechanisms, the core signaling pathways were delineated with the annotation of the KEGG pathway. The core signaling pathways of the two groups of normal cells (lean and obese), lean PCa, and obese PCa are shown in Figures S9–S12. Their corresponding gene enrichment analysis results are shown in Tables S2–S5. After comparing the core signaling pathways of lean PCa in Figure S11 and normal prostate cells (lean) in Figure S9, we could investigate the carcinogenic mechanisms of lean PCa (Figure S13). In the same way, the carcinogenic mechanisms of obese PCa could be found (Figure S14) by comparing the core signaling pathways of obese PCa (Figure S12) and normal prostate cells (obese) in Figure S10. After summarizing, we could illustrate the common and specific core signaling pathways for lean and obese PCa (Figure 2). The common and obesity-specific biomarkers of PCa were then identified based on the carcinogenic mechanism analyses. Utilizing the database for annotation, visualization, and integrated discovery (DAVID), we performed a gene enrichment analysis in the core GWGEN for normal PCa in the lean group, normal PCa in the obese group, lean PCa, and obese PCa (Tables S2–S5, respectively). Moreover, the drug discovery targeting the identified biomarkers is based on the drug design specifications. It considers drug–target interaction, drug regulation ability, and drug toxicity. One DNN-based DTI model was constructed in advance for predicting the candidate drugs with higher interaction ability with identified biomarkers. The number of predicted candidate drugs decreased after passing through drug regulation ability and drug toxicity filters. Consequently, two multiple-molecule drugs were suggested for preventing PCa (covering lean and obese) and obesity-specific PCa, respectively. Details are discussed in the following sections.

### 2.2. The Common Carcinogenic Molecular Mechanism between Lean and Obese PCa

The core signaling pathway, which is related to the immune response, is induced by receptor NOD1, gradually weakening immunity. Immune microenvironment factor IE-DAP is received by receptor NOD1, resulting in the phosphorylation of downstream molecules, which initiates chronic inflammation [20]. From the perspective of the carcinogenic mechanism for inhibiting immune response triggered by the activation of receptor NOD1 (see Figure 2), the sequential phosphorylation of proteins KLK4 [21] and COX2 are found. This gives the protein COX2 an intermediate role in PCa. Overexpression of protein COX2 could promote tumor progression, e.g., cell proliferation, angiogenesis, suppression of immune response, and enhanced metastasis [22]. Owing to the upstream phosphorylation, protein FOXB1 is activated with attenuated E-cadherin [23], which strengthens the overexpression of the downstream target gene *NANOG* in a metastasis-related pathway triggered by the receptor SORT1. Furthermore, we find that the immune response has a strong correlation with driving migration and invasion in obese PCa. Subsequently, the protein MYB is promoted, exhibiting a strong malignant phenotype. It indirectly causes cell cycle arrest in tumor cells. Once moving towards the worsening direction of cell proliferation, this aggressive feature not only plays a role of strong resistance in the treatment of drug resistance but also improves the motility of cancer cells, leading to EMT effects. Consequently, the mechanism of castration resistance, affected continuously by activation of the protein MYB, could enhance the invasive capability of both lean and obese PCa. In addition, the activation of the NOD1 receptor induced by IE-DAP could upregulate TF STAT1, acting as an active transcription factor to boost tumor metastasis in PCa and upregulate the target gene CD3G [24]. Eventually, under the overexpression of target gene CD3G, this indirectly results in the chronic inflammation of prostate tumors. Moreover, PCa cells could proliferate in the immunosuppressed microenvironment [25].

The most relevant pathway of cell proliferation is the invasive signaling pathway triggered by receptor FZD10, which is activated by ligand SS18–SSX2 in order to transduce the Wnt-11 signal [26]. In Figure 2, the microenvironment factor SS18–SSX2, related to the invasive signaling pathway, is received by receptor FZD10. It could upregulate TF SNW1 by signaling transduction proteins RBM25 and MYB, and by the activation of TF ESF1. Its target gene TMPRSS2 is upregulated, giving rise to cell proliferation, differentiation, angiogenesis, and indirectly causing an excessive inflammatory response. Consequently, the overexpression of target gene TMPRSS2 plays an important role in promoting the progression of metastatic prostate cancer. On the other hand, ATF2 protein is activated through the same signaling transduction proteins RBM25 and MYB to inhibit TF RBMX. Moreover, with the low expression of TF RBMX, it could upregulate miRNA MIR222 for the purpose of downregulating the downstream target genes DIRAS3 and DCTN6, enhancing cancer cell proliferation, cell survival rate, and inducing invasion. The inhibition of protein RBM25 is also influenced by the strongest induction effect from both the upstream signal and the downregulation of upstream TP53 suppressor in another invasive signaling pathway, for the purpose of activating protein MYB [27]. Then, the activity of protein MYB demonstrates a strong malignant phenotype in prostate tumors [28]. Most important of all, it could trigger the progression of PCa cell proliferation. Furthermore, TF ESF1 could not only upregulate target gene TMPRSS2, known as the fusion gene, via the upregulation of TF SNW1 but also specifically upregulate miRNA MIR193A in the signaling pathway of lean PCa (see Figure 2). The overexpression of TF SNW1 participates in androgen receptor splicing and transcription control, and is regarded as a co-activator of nuclear receptors [29]. Finally, influenced by the above effects, the target gene TMPRSS2, expressing a type of transmembrane protein, is overexpressed, resulting in the carcinogenic mechanism. This specific androgen response gene TMPRSS2 not only contributes to cell proliferation, differentiation, angiogenesis, inflammation, and anti-apoptotic effects but also dysregulates the body’s immune response, which enhances the late invasive ability of PCa [30]. Therefore, considering the phenomenon mentioned above, it is speculated that TMPRSS2 is a common target gene for PCa treatment. On the other hand, owing to the fact that TF RBMX, a kind of tumor suppressor, loses its function, its low expression is related to a high fructose diet and the synthesis of cholesterol [31]. Subsequently, MIR222 is upregulated through the inhibition of TF RBMX and increases the possibility of PCa recurrence, proving that MIR222 plays a significant role in obese prostate tissue [32]. Furthermore, the upregulated MIR222 induces fat formation, having a positive correlation with BMI value. Therefore, it is referred to as a key indicator for the impact of obesity on the progression of advanced PCa. Eventually, low expression of target genes DCTN6 and DIRAS3 enhance cancer cell proliferation and cell survival rate, and accelerate cancer cell invasion [32]. Overall, the upregulation of MIR222 could not only inhibit the expression of tumor suppressors such as target genes DIRAS3 and DCTN6 in prostate cancer but also prove that obesity has a strong correlation with cancer metastasis.

The most relevant pathway for EMT and metastasis is driven by the metabolic signaling pathway stimulated by receptor GALR2, as shown in Figure 2. In the obese prostate tumor, when receptor GALR2 receives a high concentration of galanin ligand, the galanin activates receptor GALR2 to increase human food intake and indirectly promote fat intake [33]. Furthermore, galanin is capable of regulating the related nerve conduction; a high concentration could trigger the absorption of fat. Through signaling transduction proteins ENPP5 and AMMECR1, transmitted into the nucleus, the signal not only suppresses miRNA MIR130B but also promotes miRNA MIR217, leading to the low expression of target gene *FOXF2* [34]. *FOXF2*, known as a tumor suppressor, is downregulated in the epithelial mesenchyme to destroy the differentiation effect. Moreover, the mesenchymal transcription factor *FOXF2* could make EMT more vigorous. On the other hand, TF SIM2 is upregulated through sequentially regulated proteins for the purpose of enhancing the invasive ability of PCa [35]. Finally, the upregulation of target gene FOXM1 resulting from overexpression of TF SIM2 cooperates with target gene *FOXF2* to accelerate the process of cell proliferation and strengthen EMT [36]. In addition, influenced by the activation of receptor GALR2, TF MYOCD is upregulated after the signal is transmitted into the nucleus, which causes the development and differentiation of smooth muscle, indirectly promoting the migration of cancer cells [37]. Finally, target gene *FOXF2* is inhibited through the overexpression of miRNA MIR182, also due to the activation of TF MYOCD [38].

The common pathway of metabolism is induced by receptor TMEM123, a kind of transmembrane protein. When receiving the protein-complex ligand, as shown in Figure 2, the highly glycosylated TMEM123 receptor could not only lose the capability of triggering tumor cell apoptosis but also activate downstream protein SYMPK [39,40]. In addition to influencing the downstream lipid metabolism, this signaling transmission induced by protein SYMPK could suppress protein SAMD2, in order to make the apoptotic mechanism abnormal after entering the nucleus. Moreover, the repression of protein SMAD2 could lead to malignant transformation of tumors owing to abnormal expression of genes related to cancer cell apoptosis [41]. Then, as a crucial tumor suppression factor of PCa epithelium, protein SMAD2 is inhibited, resulting in TF ESF1 upregulation and oxidatively induced DNA damage. As a result of the overexpression of TF ESF1, miRNA MIR133A1 is downregulated to upregulate the target gene *EGFR*, responsible for cell proliferation, local recurrence, and distant-organ metastasis in prostate tumors [34].

In summary, according to our investigation of carcinogenic mechanisms, PCa suffers not only from the suppression of apoptosis, immune response, and metabolism but also from the stimulation of proliferation, EMT, and metastasis. Furthermore, in the common signaling pathway shown in Figure 2, the phosphorylated protein COX2 plays the role of mediator, and can accept the activation response of phosphorylation of CERK from the metabolic signaling pathway. It is indicated that the metabolic response is closely related to the secretion of chemokines and cytokines in the immune response, further influencing the migration ability of tumor cells. That is, the overall enhancement of ability in cell proliferation, angiogenesis, and anti-apoptosis proves that COX2 is a key driving gene. Therefore, overexpression of target gene CD3G could inhibit the immune response and gradually destroy the immune system in the human body via chronic inflammation, building a bridge between immune response and metabolism. This phenomenon explains how, in the incubation period of tumors in the early stage of PCa, tumors can resist the immune system of the host. Consequently, we took *FOXF2*, *EGFR*, MYB, SIM2, STAT1, and SMAD2 as biomarkers. We aimed to restore them to their normal expression levels via drug discovery and design. Generally, *FOXF2* and *EGFR* are associated with proliferation and anti-apoptosis in cancer, showing that the *FOXF2* gene plays a critical role in tumor suppression. MYB not only reduces the motility of cancer cells but also slows down the secretion of cytokines and chemokines by cancer cells, showing a strong malignant phenotype in prostate tumors. The upregulation of TF SIM2 stimulates the activity of downstream oncogene FOXM1 to construct an appropriate microenvironment for cancer cell metastasis through the secretion of exosomes from the malfunction of metabolism. The unphosphorylated state of TF STAT1 enduring upregulation is responsible for the promotion of cancer growth and metastasis, and the deterioration of the immune response, leading to drug resistance and forcing cancer cells to invade and migrate to other organs in advanced PCa. The inhibition of SMAD2 protein in the highly glycosylated signaling pathway stimulated by receptor TMEM123 can regulate the anti-apoptosis ability of cancer cells.

### 2.3. The Specific Molecular Mechanism in Lean PCa

In the specific core signaling pathway of apoptosis in Figure 2, with high concentration of growth hormone (GH) in the microenvironment of lean PCa, receptor GHR receives GH ligands to repress the downstream AKT signaling pathway, stimulating GHR itself. Due to the high concentration of GH in lean PCa cells, it will regulate somatic cell growth and substrate metabolism, preventing the cancer cells from being prone to local autocrine and paracrine effects [42]. Additionally, it will also inhibit the capability of interacting with androgens or growth factors, leading to the downstream suppression of proliferation and the promotion of apoptosis in tumors. Then, through the signaling transmission of cascade proteins DNAH14 and CPSF4, it could enter the nucleus to downregulate TF MED17, resulting in an androgen-dependent reduction of cancer cells [43]. Inhibited by TF MED17, target gene DNMT1 undergoes a DNA demethylation reaction, not only weakening its carcinogenicity but also contributing to a gradual slowdown of the cell proliferation rate and a promotion of apoptosis, which plays a crucial role prior to tumor cell metastasis [44].

In another specific core signaling pathway related to the immune response (see Figure 2), the ligand WNT5A in the lean PCa microenvironment is relevant to the signal transduction of lipid modification and the glycoprotein. Moreover, WNT5A plays a different kind of role in normal cells (lean) and lean PCa, depending on its concentration. When receptor ROR2 binds to a small amount of WNT5A, it could slow down the secretion of cytokines and chemokines from cancer cells [45], regulating the mutated LRSAM1. Influenced by the secreted signal of lipid-modified glycoprotein, the dysregulation of protein LRSAM1 could inhibit downstream protein MYB, indirectly reducing the motility of cancer cells. Furthermore, the inhibition of protein HOXB13 downregulates TF ESF1, to stabilize the ability to repair oxidatively induced DNA damage [46]. It is noted that miRNA MIRLET7A1 is upregulated to inhibit the expression of target gene IGF1R [47]. Once target gene IGF1R is downregulated, it could trigger the metabolism for the purpose of accelerating cancer cell apoptosis and inhibiting cancer cell proliferation. At the same time, the inhibition of target gene EZH2 plays a role as a cell cycle regulator, inhibiting cancer cell proliferation [34]. Therefore, we infer that this immune-like signaling pathway plays a significant role in inducing downstream metabolism.

### 2.4. The Specific Carcinogenic Molecular Mechanism in Obese PCa

In one specific core signaling pathway related to metabolism shown in Figure 2, receptor OSBPL2 combines with sterols and a microenvironment factor PI4P to regulate cell proliferation signals [48]. Obesity will trigger PI4P ligands to inhibit the expression of receptor OSBPL2, resulting in the accumulation of fat and cholesterol in the downstream signaling pathway. Then, the activation of protein VCAM1 induced by receptor OSBPL2 could result in PCa easily acquiring aggressive characteristics. In other words, distant metastasis is more likely to occur [49]. It is speculated that obesity is a critical factor in the advanced malignant phenotype of PCa. Subsequently, protein CERK is phosphorylated to induce the synthesis of ceramide, including the main components of lipids, cholesterol, and fatty acids, which form a lipid bilayer structure [50]. The activation of conduction factor CERK indirectly triggers dysregulation of the immune response, cancer cell proliferation, and metastasis by upregulating TF MYBL2 to overexpress. When enhancing the adhesion ability of the extracellular matrix (including laminin, collagen, and fibronectin), TF MYBL2 is upregulated to promote the activity of epithelial–mesenchymal transition (EMT), enabling tumor cells to acquire the characteristics of migration and invasion [51]. As a result of the decreased expression of cadherin E, TF MYBL2 upregulates target gene IRS2 to be phosphorylated and overexpressed, which stimulates IRS2 to interact with membrane insulin tyrosine kinase receptors resulting in activating the downstream Ras/mitogen-activated protein kinase (MAPK) pathway [52]. Consequently, the overexpression of target gene IRS2 may disrupt the secretion of insulin, promote cell growth, and repress the normal metabolism of the human body.

In the specific core signaling pathway concerning immune response shown in Figure 2, receptor TLR4 generally stimulates its own anti-tumor immune signaling pathway without binding the IL1B ligand, triggering immune surveillance in the tumor microenvironment with the mechanism of allowing macrophages to play a pro-apoptotic role [53]. Then, it could promote DNA hypomethylation of protein TOLLIP, negatively stimulating its expression. Nevertheless, there is an opposite effect in the same signaling pathway in obese PCa, as shown in Figure 2. The anti-tumor immune signaling pathway is inhibited when receptor TLR4 is bound by the IL1B ligand in the microenvironment. Furthermore, because of the negative correlation between expression of the protein TOLLIP and DNA methylation, sex steroid hormones could stimulate an inflammatory response by the inhibition of the protein TOLLIP [54], that is, the hypermethylation of these hormones activates the expression of proinflammatory cytokines, which gives rise to downstream phosphorylation of protein STAT3 [55] (see Figure 2). Subsequently, TF MYC is overexpressed through high expression of protein STAT3. As well as driving the formation of prostate intraepithelial neoplasia [56], overexpression of TF MYC could further suppress target gene HSH2D. Since target gene HSH2D plays an important role in T cell activation, its low expression could reduce the activity of anti-tumor T cells [57]. It is speculated that this immune-like pathway occurs particularly in obese patients of PCa, and the metabolism-related pathway induced by receptor OSBPL2 plays a significant role in interacting with it.

In the specific core signaling pathway concerning metastasis shown in Figure 2, once the microenvironment factor PCSK9 binds to receptor SORT1 to drive itself to be activated [58], it could not only stimulate protein CERK, making it phosphorylated, but also induce downstream mutation of protein TP53. Through the activation of protein CERK, it could stimulate the neurotensin signal to produce excessive fatty acid, resulting in obesity. Therefore, it is proved that this invasive signaling pathway indirectly leads to an accumulation of excess fatty acids and the production of cholesterol, making obese PCa capable of malignant metastasis. Then, when protein TP53 is mutated and further suppressed to a low-expression state, it could play an indispensable role in EMT before activating metastasis procedures [27]. It is reported that this suppressive gene is particularly common in advanced PCa. Subsequently, the inhibition of protein TP53 could promote protein OSR2 for the purpose of strengthening the ability for castration resistance and increasing the treatment difficulty of androgen deprivation [59]. After protein OSR2 is activated, it could trigger downstream TF NKX2-5 to be highly methylated and overexpressed, resulting in a methylation frequency higher than the mutation rate [33]. Apart from being a characteristic of advanced PCa, methylation of NKX2-5 upregulates target gene *NANOG* to influence the regulatory function of androgen receptors, disrupting the secretion system [60]. Finally, the overexpression of target gene *NANOG* is a significant indicator of castration-resistant prostate, contributing to cell proliferation, cancer cell regeneration, induction of the shortened cell cycle, severe invasiveness, and cancer metastasis. Additionally, the metastasis-related signaling pathway induced by receptor FZD10 is enhanced by the mutation of upstream protein TP53 and the capability for tumor metastasis is strengthened through the signaling pathway of chronic inflammation triggered by receptor NOD1.

To summarize, according to the cooperative reaction of two pathways induced by activation of receptors SORT1 and OSBPL2 (see Figure 2), obesity can drive malignant obese PCa. The overall carcinogenic mechanisms of obese PCa include the promotion of metabolism and metastasis, and the inhibition of the immune response. Finally, STAT1, *FOXF2*, SIM2, SMAD2, CERK, STAT3, and TP53 were selected as essential biomarkers (drug targets) for obese PCa.

### 2.5. The Application of Deep Neural Network to Drug–Target Interaction Prediction and the Drug Design Specifications Considering Drug Regulation Ability and Drug Toxicity

**Figure 3:**
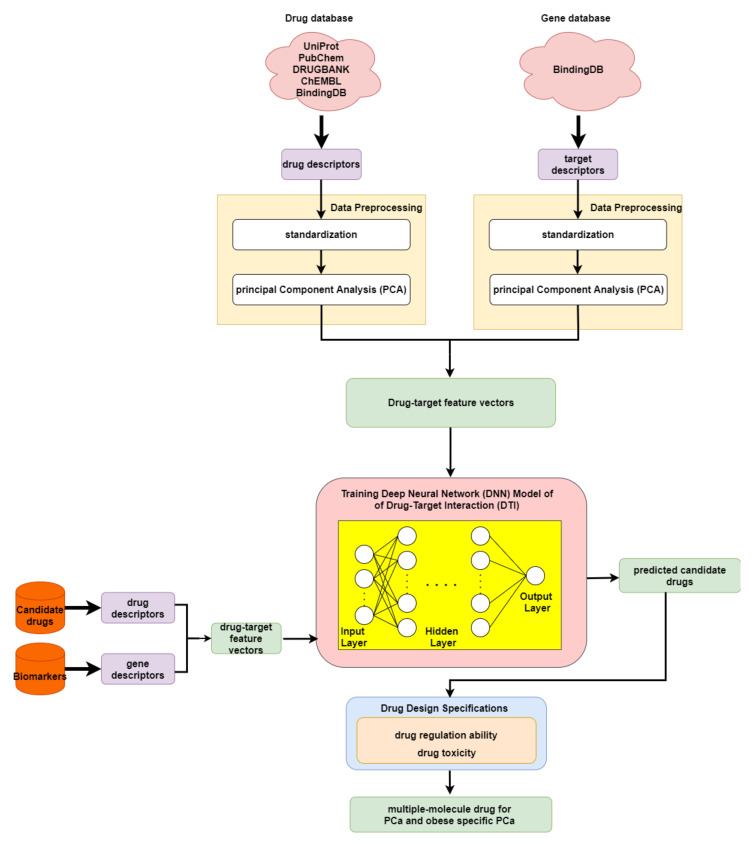
Flowchart of drug discovery method for multiple-molecule drug design.

In order to predict the drug–target interaction probability for our identified biomarkers, we trained a DNN-based DTI model in advance for drug–target interaction prediction. Subsequently, we introduced drug regulation ability and drug toxicity into our drug design specifications. The whole drug design flowchart, including one DNN-based DTI model, can be seen in Figure 3. The interaction dataset used for training was from BindingDB [61]. In total, there are 80291 known drug–target interactions between 38015 drugs and 7292 proteins. The number of unknown drug–target interactions is 19966109. To avoid the class imbalance issue, we randomly chose a set of unknown interactions to be the same size as the known interactions. We trained the model using 70% of the data, including 10% of the data as a validation set. The remaining 30% of the data were used as the testing set. Before training the DNN-based DTI model, we performed feature scaling by standardization. Then, PCA was adopted to perform the dimension reduction, giving 694 out of 1359 features. For the architecture of the DNN-based DTI model, we used Adam as an optimizer (learning rate = 0.0001) with binary cross-entropy loss. The input layer had 694 nodes followed by 512, 256, 128, and 64 nodes in the four hidden layers, respectively. The output layer had one node. We used the sigmoid function in the output layer and set the nonlinear activation function ReLU for each hidden layer. Furthermore, dropout was added to each hidden layer for reducing overfitting. Meanwhile, early stopping was used to terminate the model training once the model performance stopped improving on the validation set. Here, we applied 10-fold cross validation to evaluate the model performance, as shown in Table S6. The learning curve for the 10-fold cross validation is shown in Figure S16a,b. Finally, the average accuracy for the 10-fold cross validation was 94.89% (standard deviation: 0.131). It is noted that the model with the best testing accuracy was reserved for making drug–target interaction predictions for our identified biomarkers. The area under the receiver operating characteristic curve (AUC) is useful for organizing binary classifiers and visualizing their performance. The AUC of the reserved model was 0.99, as shown in Figure S17.

In order to narrow down the range of candidate drugs predicted by the DNN-based DTI model, we took drug regulation ability and drug toxicity into account in our drug design specifications. Furthermore, we aimed to find compounds that had the ability to target multiple identified drug targets. For drug regulation ability, we referred to the library of integrated network-based cellular signatures (LINCS) L1000 level 5 dataset [62]. We could identify whether a gene expression was up or downregulated after treatment with the small-molecule compound. Our goal here was to reverse the abnormal gene expression for our identified biomarkers. For drug toxicity, we looked up the median lethal dose (LD50) in DrugBank for our candidate drugs. We aimed to find drugs that not only could reverse the abnormal gene expression but also had higher LD50 values. In addition, drugs with lower toxicity have fewer side effects.

## 3. Discussion

### 3.1. Systems Biology Approaches and Traditional Treatments for PCa

By leveraging systems biology approaches, we investigated differential core signaling pathways with carcinogenic molecule mechanisms for PCa. Compared with normal PCa (lean and obese groups), for lean PCa and obese PCa we focused on the common and specific core signaling pathways induced by microenvironmental factors, which further trigger downstream target genes to exert effects on cellular functions including metabolism, immune response, metastasis, epithelial–mesenchymal transition (EMT), apoptosis, and cell proliferation. The common and specific core signaling pathways (Figure 2) have been discussed in detail, based on their corresponding cellular functions. Moreover, the supervised learning method was employed to construct a DNN-based DTI model. In terms of the application, we used this to help us predict potential candidate drugs that had a higher interaction probability with our identified biomarkers. Following the drug design specification, we further considered drug regulation ability and drug toxicity. Finally, we suggested two multiple-molecule drugs for PCa (covering lean and obese PCa) and obesity-specific PCa after filtering the candidate drugs shown in Table S7 based on the drug design specifications.

Nowadays, there are few drawbacks in traditional treatments including active surveillance, surgery, and radiation therapy in patients with PCa. For low-risk PCa, active surveillance methods such as monitoring PSA level, repeat biopsies, or determining the Gleason grade are not suitable for men with other low-risk diseases that cause side effects [63]. If patients have other organ problems, active surveillance could not only potentially result in general health changes but also cause severe discomfort or pain to patients. In addition, after radical prostatectomy surgery, it may increase the chance of erectile dysfunction and a large number of complications such as blood clots, a reaction to the medicine, or an infection [64]. If surgery is conducted after radiation therapy, it could elevate the incidence of wound complications and poor healing. Without killing all the cancer cells in tumors, radiation therapy leads to damage to surrounding tissues, according to the distance between the area of interest and the prostate tumor, resulting in further patient fatigue due to the energy consumption used to kill normal cells in the procedure [65]. Moreover, from the point of view of using molecular drugs, there are some obstacles to treatments using a single drug for a single target in a single disease. For example, bicalutamide is commonly known as a selective antagonist towards the androgen receptor (AR), which plays a role in targeting androgens such as DHT and testosterone. Although it was found to accelerate the degradation of AR, it has estrogenic effects, inducing gynecomastia for men with monotherapy, paradoxically stimulating the occurrence of late-stage PCa. Therefore, in this study, we attempted to find multiple-molecule drugs that could influence multiple identified biomarkers. Additionally, it is noted that drug repositioning methods are gradually becoming spotlighted due to the short time frame for drug development and low cost. The systems biology approach provides an alternative method for exploring new therapeutics for PCa and obesity-specific PCa.

### 3.2. Multiple-Molecule Drugs for PCa and Obesity-Specific PCa

We have taken advantage of systems biology methods to recognize significant biomarkers as indispensable drug targets (see Table 1) for PCa (covering lean and obese) and obesity-specific PCa, individually. Moreover, these drug targets were chosen based on the carcinogenic molecular mechanisms. The systems drug discovery method involves designing multiple-molecule drugs to restore the abnormal expression of drug-targeted genes to their normal expression, with higher drug–target interaction probability and lower drug toxicity. According to the results predicted by the DNN-based DTI model, we firstly obtained candidate drugs with higher interaction probability with our identified biomarkers. Subsequently, the range of candidate drugs was narrowed down by considering drug regulation ability using CMap and drug toxicity using a median lethal dose (LD50) filter. Generally, LD50 is used as reference during drug discovery, since lower drug toxicity is associated with reduced side effects. A drug with a lower LD50 value is more toxic. Gathering the results of the drug design specifications, including drug–target interaction, drug regulation ability, and drug toxicity for the identified biomarkers shown in Table S7, we suggested two multiple-molecule drugs for PCa (covering lean and obese) and obesity-specific PCa, respectively.

In Table 2 and Table 3, we observe that apigenin has six drug targets, including STAT1, SIM2, *EGFR*, MYB, CERK, and STAT3. Apigenin, a nutraceutical drug, plays a significant role in tumor suppression efficacy and in preventing a large number of chronic diseases, especially diabetes. More and more evidence has demonstrated that apigenin functions as a promising therapeutic anti-inflammatory, presenting properties with anti-tumor efficacy against various types of tumors. In PCa, it has been reported in clinical trials that apigenin could target STAT1 as an inhibitor to stimulate the activity of T cells and immune surveillance, further recovering the patient’s own immune-system-associated cell-death genes [66]. As a natural bioactive flavone-type molecule, it has been claimed that apigenin shows pro-health properties, including stimulating immune-like pathways, inhibiting cell proliferation by inducing apoptosis, and suppressing metastasis. Therefore, we considered apigenin as one of the constituents of our potential multiple-molecule drug, to slow down cancer cell motility and decrease the occurrence of severe invasion in obese PCa [67]. This result provides a novel perspective for cancer immunotherapy in preventive healthcare. Interestingly, digoxin, a cardiac glycoside, was chosen as a constituent in our potential multiple-molecule drug, for drug targets containing *FOXF2*, SMAD2, CERK, and TP53. Digoxin can influence the properties of the sodium potassium channel on the plasma membrane to alter the calcium ion concentration, enhancing apoptosis of cancer cells. Moreover, for patients not receiving androgen deprivation therapy (ADT) as the primary treatment for PCa, digoxin is a promising therapeutic agent exerting protective effects on anti-tumor characteristics [68]. It was also reported that digoxin could significantly suppress MYC expression and elevate SMAD2 expression to induce apoptosis, revealing its vigorous cytotoxic effects in regulating apoptosis-related signaling pathways, with the benefit of low drug toxicity in tumors [69]. Additionally, there is a significant anti-obesity drug, orlistat, that has a tremendous drug effect on inhibiting fatty acid synthase. With fewer side effects, moderate toxicity, and exceptional regulation ability, orlistat, combined with other anti-proliferative drugs, could exert its influence on the vital increase in apoptosis and the suppression of tumor cell viability and proliferation. Through blocking the synthesis of the vast majority of fatty acids in the tumor microenvironment, orlistat favors the boosting of the metabolism to impede the efforts of the cell energy machinery to stimulate proliferation, demonstrating that it is a promising therapeutic agent for obese PCa [70].

In summary, two multiple-molecule drugs for PCa (covering lean and obese) and obesity-specific PCa are suggested, as shown in Table 2 and Table 3, respectively. Apigenin has the ability to inhibit MYC in obese PCa by downregulating STAT3. It is noted that apigenin does not drive the formation of prostate intraepithelial neoplasia and suppresses MYB to indirectly reduce the mobility of cancer cells, to avoid metastasis and cell proliferation for lean PCa. Most important of all, apigenin plays a role in triggering the immune response by activating T cells. Digoxin is beneficial in resisting the malignant transformation of tumors through the upregulation of SMAD2, inducing apoptosis for PCa. In addition, orlistat not only helps in suppressing malignant metastasis procedures but also favors the strengthening of the human immune response by dephosphorylating CERK to reduce the synthesis of ceramide, resulting in less fatty acid and cholesterol production.

## 4. Materials and Methods

### 4.1. A General Review of Constructing Core Genome-Wide Genetic and Epigenetic Networks (GWGENs) of Normal Prostate Cells, and Lean and Obese PCa

In order to perform a comprehensive analysis of molecular mechanisms in PCa and discover the common and specific molecular mechanisms, we needed to fully compare four core genome-wide genetic and epigenetic networks (GWGENs). Adopting a BMI of 25 as the obesity threshold, the data were split into two groups: one for lean people and the other for obese people. With the benefit of systems biology [71], we can further extract the four core signaling pathways of normal prostate cells (including lean and obese groups), and lean and obese PCa from their core GWGENs. The methods for finding the core signaling pathways from the candidate GWGEN can be seen in the flowchart in Figure 1. Here, we split the process into five steps as follows:

(1) Constructing the candidate GWGEN. Using big database mining, we constructed a candidate PPIN and a candidate GRN, including genes, miRNA, and lncRNA, as the first step. It is noted that the candidate GWGEN consists of a candidate PPIN and a candidate GRN.
(2) Identifying real GWGENs. After performing system modeling for proteins, genes, miRNA, and lenRNA, we performed system identification by solving the constrained linear least squares estimation problem with the help of the microarray data for normal prostate cells (including lean and obese groups), and lean and obese PCa. We then used the system order detection scheme for computing the AIC, to prune the false-positive interactions in the candidate GWGEN.
(3) Extracting the core GWGENs. To extract the core GWGENs, we applied the PNP approach. By doing so, we could compute a projection value for each node in the real GWGENs. The top 3000 elements with highest projection values remained.
(4) Building and comparing the core pathways. The core signaling pathways for normal prostate cells (including lean and obese groups), and lean and obese PCa in the annotation of KEGG pathways could be found by referring to the projection values and the literature survey. We investigated the molecular mechanisms of carcinogenesis considering the microenvironmental factors of lean and obese PCa and their corresponding downstream core signaling pathways.
(5) Identifying biomarkers (drug targets) for the design of multiple-molecule drugs. Based on the analysis of carcinogenic molecular mechanisms, we identified essential biomarkers for PCa (covering lean and obese) and obesity-specific PCa. Following the proposed drug design specifications, we considered drug–target interaction probability, drug regulation ability, and drug toxicity. One DNN-based DTI model was trained in advance for predicting candidate drugs targeting identified biomarkers. The aim of the drug regulation ability filter was to reverse the abnormal expression of biomarkers. The drug toxicity filter helped to find drugs with light toxicity. Consequently, we suggested two multiple-molecule drugs for PCa (covering lean and obese) and obesity-specific PCa.

### 4.2. Data Preprocessing for Constructing the Candidate GWGEN

In this study, we downloaded the dataset with accession number GSE79021 from the National Center for Biotechnology Information (NCBI). The samples in the dataset were divided into four groups, containing normal prostate cells (lean and obese groups), and lean and obese PCa. We only used samples that had BMI information. Hence, we had 49 samples for normal prostate cells and 153 samples for PCa. Subsequently, we individually classified the normal prostate cells and PCa cells into two categories using the body mass index (BMI, threshold: 25) for investigating the influence of obesity on PCa. In this way, 25, 24, 70, and 83 samples were obtained for normal prostate cells in the lean group, normal prostate cells in the obese group, PCa in the lean group, and PCa in the obese group, respectively. It is noted that the candidate GWGEN comprised a candidate PPIN and a candidate GRN. Therefore, it was a binary matrix. If two nodes have an interaction, we assigned a value of one, otherwise we assigned a value of zero. For building the candidate PPIN, we referred to the following databases: DIP [72], IntAct [73], BioGRID [74], and MINT [75]. Moreover, to construct the candidate GRN, we considered the following databases: HTRIdb [76], ITFP [77], TRANSFAC [78], CircuitDB2 [79], TargetScanHuman [80], and starBase 2.0 [81].

### 4.3. System Modeling for Normal Prostate Cells and PCa

After constructing the candidate GWGEN, we performed system modeling for proteins, genes, miRNA, and lncRNA [82]. We can describe the *i*-th protein in the candidate PPIN by the following equation:

(1) $$\begin{array}{ll} p_{i}[n]=\sum_{\begin{array}{l} h=1\\ h\neq i \end{array}}^{H_{i}}\alpha_{ih}p_{i}[n]p_{h}[n] & +\lambda_{i,PPI}+\phi_{i,PPIs}[n],\\ & \;\mathrm{for}\;i=1,\ldots ,I\;,\;n=1,\ldots ,N \end{array}$$

where $p_{i}[n]$ and $p_{h}[n]$, respectively, denote the expression level of the *i*-th and *h*-th protein for the n-th sample; $\alpha_{ih}$ denotes the interaction ability between the *i*-th protein and the *h*-th protein; $H_{i}$ indicates the total number of proteins interacting with the *i*-th protein; I indicates the total number of proteins in the candidate PPIN; N represents the total number of data samples; and $\lambda_{i,PPI}$ expresses the basal level of the i-th protein. The change in basal level is related to post-translational modification, including phosphorylation, acetylation, and methylation. In addition, $\phi_{i,PPIs}[n]$ shows the stochastic data noise of the *i*-th protein.

Similarly, the *j*-th gene in the candidate GRN, which is part of the candidate GWGEN for sample *n* is given as shown below:

(2) $$\begin{array}{ll} g_{j}[n]=\sum_{\begin{array}{l} r=1\\ r\neq j \end{array}}^{R_{j}}a_{jr}T_{r}[n]+\sum_{s=1}^{S_{j}}b_{js}L_{s}[n] & \;-\;\sum_{u=1}^{U_{j}}c_{ju}M_{u}[n]g_{j}[n]+\lambda_{j}+\phi_{j}[n],\;\\ & \;\mathrm{for}\;j=1,\ldots ,J\;,\;n=1,\ldots ,N \end{array}$$

where $g_{j}[n]$ indicates the expression level of the *j*-th gene; $a_{jr}$ and $b_{js}$ individually represent the transcription regulatory ability from the *r*-th TF and the *s*-th lncRNA to the *j*-th gene; $-c_{ju}\leq 0$ denotes the post-transcription regulatory ability by which the *u*-th miRNA restrains the j-th gene; $R_{j}$ and $S_{j}$, respectively, denote the total number of TFs and lncRNAs binding to the *j*-th gene; $U_{j}$ indicates the total number of miRNAs inhibiting the *j*-th gene; and $T_{r}[n]$, $L_{s}[n]$, and $M_{u}[n]$ individually denote the expression of the *r*-th TF, the *s*-th lncRNA, and the *u*-th miRNA. N and J are respectively the total number of data samples and genes; $\lambda_{j}$ indicates the basal level of the *j*-th gene expression; and $\phi_{j}[n]$ represents the stochastic data noise of the gene expression in the *j*-th gene for the sample *n*. The system modeling for miRNA and lncRNA is described in the Supplementary Materials.

### 4.4. Utilizing System Identification and System Order Detection Methods to Identify Real GWGENs from the Candidate GWGEN 

So far, we have formulated each protein, gene, lncRNA, and miRNA in the candidate GWGEN. Further, with the help of the microarray data, we estimate the regulation parameters shown in Equations (1) and (2). Hence, firstly, we can rewrite Equations (1) and (2) as follows:

(3) $$p_{i}[n]=\left[p_{i}[n]p_{1}[n]\cdots p_{i}[n]p_{H_{i}}[n]\;1\right]\times \left[\begin{array}{c} \alpha_{i1}\\ \vdots \\ \alpha_{iH_{i}}\\ \lambda_{i} \end{array}\right]+\phi_{i}[n]$$

(4) $$g_{j}[n]=\left[T_{1}[n]\cdots T_{R_{j}}[n]\;L_{1}[n]\cdots L_{S_{j}}[n]\;g_{j}[n]M_{1}[n]\cdots g_{j}[n]M_{U_{j}}[n]\;1\right]\times \left[\begin{array}{c} a_{j1}\\ \vdots \\ a_{jR_{j}}\\ b_{j1}\\ \vdots \\ b_{jS_{j}}\\ -c_{j1}\\ \vdots \\ -c_{jU_{j}}\\ \lambda_{j} \end{array}\right]+\phi_{j}[n]$$

For simplicity, Equations (3) and (4) can be represented by the following linear regression forms, respectively:

(5) $$p_{i}[n]=\beta_{i,P}[n]\cdot \gamma_{i,P}+\phi_{i}[n],\;\mathrm{for}\;i=1,\ldots ,I,\;n=1,\ldots ,N$$

(6) $$g_{j}[n]=\beta_{j,G}[n]\cdot \gamma_{j,G}+\phi_{j}[n],\;\mathrm{for}\;j=1,\ldots ,J,\;n=1,\ldots ,N$$

where $\gamma_{i,P}$ indicates the parameter vector related to the protein–protein interaction abilities; $\gamma_{j,G}$ represents the parameter vectors, including the regulation abilities of TFs and the post-transcriptional regulatory abilities of lncRNAs and miRNAs; and $\beta_{i,P}[n]$ and $\beta_{j,G}[n]$ separately denote the expression vectors of the protein and gene for sample *n*.

Since we have N samples, Equations (5) and (6) can be augmented into the following equations:

(7) $$\left[\begin{array}{c} p_{i}[1]\\ p_{i}[2]\\ \vdots \\ p_{i}[N] \end{array}\right]=\left[\begin{array}{c} \beta_{i,P}[1]\\ \beta_{i,P}[2]\\ \vdots \\ \beta_{i,P}[N] \end{array}\right]\cdot \gamma_{i,P}+\left[\begin{array}{c} \phi_{i}[1]\\ \phi_{i}[2]\\ \vdots \\ \phi_{i}[N] \end{array}\right]\;$$

(8) $$\left[\begin{array}{c} g_{j}[1]\\ g_{j}[2]\\ \vdots \\ g_{j}[N] \end{array}\right]=\left[\begin{array}{c} \beta_{j,G}[1]\\ \beta_{j,G}[2]\\ \vdots \\ \beta_{j,G}[N] \end{array}\right]\cdot \gamma_{j,G}+\left[\begin{array}{c} \phi_{j}[1]\\ \phi_{j}[2]\\ \vdots \\ \phi_{j}[N] \end{array}\right]$$

In addition, the above equations can be represented as follows:

(9) $$P_{i}\;=\;\varepsilon_{i,P}\cdot \gamma_{i,P}\;+\;\varphi_{i}$$

(10) $$G_{j}\;=\;\varepsilon_{j,G}\cdot \gamma_{j,G}\;+\;\varphi_{j}$$

To estimate the regulatory parameters in Equations (9) and (10), we transform them into linear least squares estimation or constrained linear least squares estimation problems as below:

(11) $${\hat{\gamma }}_{i,P}=\underset{\gamma_{i},P}{\min}\frac{1}{2}{\left\|\varepsilon_{i,P}\cdot \gamma_{i,P}-P_{i}\right\|}_{2}^{2}$$

(12) $${\hat{\gamma }}_{j,G}=\underset{\gamma_{j},G}{\min}\frac{1}{2}{\left\|\varepsilon_{j,G}\cdot \gamma_{j,G}-G_{j}\right\|}_{2}^{2}$$

$$\;\mathrm{subject}\;\mathrm{to}\;\;\begin{array}{cccc} {}[\begin{array}{cccc} 0 & \cdots & \cdots & 0\\ \vdots & \ddots & & \vdots \\ \vdots & & \ddots & \vdots \\ 0 & \cdots & \cdots & 0 \end{array} & \begin{array}{cccc} 0 & \cdots & \cdots & 0\\ \vdots & \ddots & & \vdots \\ \vdots & & \ddots & \vdots \\ 0 & \cdots & \cdots & 0 \end{array} & \begin{array}{cccc} 1 & 0 & \cdots & 0\\ 0 & \ddots & \ddots & \vdots \\ \vdots & \ddots & \ddots & 0\\ 0 & \cdots & 0 & 1 \end{array} & \begin{array}{c} 0\\ \vdots \\ \vdots \\ 0 \end{array}] \end{array}\gamma_{j,G}\leq \left[\begin{array}{c} 0\\ \vdots \\ \vdots \\ 0 \end{array}\right]\;R_{j}\;\;\;\;\;S_{j}\;\;\;\;\;U_{j}$$

It is noted that (12) represents a constrained linear least squares estimation problem. By adding a matrix inequality in (12), we can guarantee that the estimated post-transcriptional regulatory abilities from miRNA are negative. Moreover, Equations (11) and (12) can be solved via the MATLAB optimization toolbox.

The candidate GWGEN is built from various databases. False-positive interactions may exist within the candidate GWGEN. Therefore, we performed system order selection by computing the AIC. According to AIC theory, the real system order would lead to the smallest AIC value [83]. The AICs for the *i*-th protein and the *j*-th gene are as given below:

(13) $$\begin{array}{l} AIC(H_{i})=\log({\hat{\hbar }}_{i,P}^{2})+\frac{2({ƛ}_{i,P})}{N}\\ \mathrm{where}\;{\hat{\hbar }}_{i,P}=\sqrt{\frac{{\left(P_{i}-(\varepsilon_{i,P}\cdot {\hat{\gamma }}_{i,P})\right)}^{T}(P_{i}-(\varepsilon_{i,P}\cdot {\hat{\gamma }}_{i,P}))}{N}}\;,\;{ƛ}_{i,P}=H_{i}+1 \end{array}$$

(14) $$\begin{array}{l} AIC(R_{j},S_{j},U_{j})=\log({\hat{\hbar }}_{j,G}^{2})+\frac{2({ƛ}_{j,G})}{N}\\ \mathrm{where}\;{\hat{\hbar }}_{j,G}=\sqrt{\frac{{\left(G_{j}-(\varepsilon_{j,G}\cdot {\hat{\gamma }}_{j,G})\right)}^{T}(G_{j}-(\varepsilon_{j,G}\cdot {\hat{\gamma }}_{j,G}))}{N}}\;,\;{ƛ}_{j,G}=R_{j}+S_{j}+U_{j}+1 \end{array}$$

where ${\hat{\hbar }}_{i,P}^{2}$ and ${\hat{\hbar }}_{j,G}^{2}$ individually denote the estimated residual error of the *i*-th protein and the *j*-th gene; ${ƛ}_{i,P}$ and ${ƛ}_{j,G}$ are the number (order) of parameters for the *i*-th protein in the parameter estimation problem in (11) and the number (order) of parameters for the *j*-th gene in the parameter estimation problem in (12), respectively; and ${\hat{\gamma }}_{i,P}$ and ${\hat{\gamma }}_{j,G}$ individually indicate the estimated parameters of the i-th protein in (11) and the estimated parameters of the j-th gene in (12). The real system order $H_{i}{}^{*}$ for the *i*-th protein and $R_{j}{}^{*},S_{j}{}^{*},U_{j}{}^{*}$ for the *j*-th gene would give the smallest AIC value. In other words, all the insignificant interactions and regulations outside the real system order are pruned for each protein, gene, lncRNA, and miRNA in the candidate GWGEN. The method of performing system identification and utilizing the system order detection scheme for lncRNA and miRNA is given in the Supplementary Materials. The real GWGENs of normal prostate cells (including lean and obese groups), and lean and obese PCa are shown in Figures S1–S4, respectively.

### 4.5. Extracting Core GWGENs from the Real GWGENs Using the Principal Network Projection Method

The real GWGEN is too complex for investigating the discrepancies in genetic and epigenetic mechanisms between normal prostate cells (including lean and obese), and lean and obese PCa. Therefore, we employed the PNP method on the real GWGEN to extract the relevant core GWGEN. Firstly, we constructed a system matrix *K*, including all estimated parameters of the real GWGEN. The system matrix *K* is:

(15) $$K=\left[\begin{array}{ccc} k_{protein↔protein} & 0 & 0\\ k_{TF\to gene} & k_{\ln cRNA\to gene} & k_{miRNA\to gene}\\ k_{TF\to \ln cRNA} & k_{\ln cRNA\to \ln cRNA} & k_{miRNA\to \ln cRNA}\\ k_{TF\to miRNA} & k_{\ln cRNA\to miRNA} & k_{miRNA\to miRNA} \end{array}\right]$$

where the sub-network matrix $k_{protein↔protein}$ denotes the system matrix of interactive abilities of proteins; the sub-network matrices $k_{TF\to gene}$, $k_{TF\to \ln cRNA}$ and $k_{TF\to miRNA}$ indicate the relevant system matrices associated with TFs transcriptional regulatory abilities for genes, lncRNAs, and miRNA, respectively; the sub-network matrices $k_{\ln cRNA\to gene}$, $k_{\ln cRNA\to \ln cRNA}$, and $k_{\ln cRNA\to miRNA}$ represent the relevant system matrices associated with lncRNAs post-transcriptional regulatory abilities for genes, lncRNAs, and miRNA, respectively; and the sub-network matrices $k_{miRNA\to gene}$, $k_{miRNA\to \ln cRNA}$, and $k_{miRNA\to miRNA}$ show the relevant system matrices associated with miRNAs post-transcriptional regulatory abilities for genes, lncRNAs, and miRNA, respectively. The detailed elements of system matrix K are shown in the following:

(16) $$K=[\begin{array}{ccc} \begin{array}{ccccc} {\hat{\alpha }}_{11} & \cdots & {\hat{\alpha }}_{1h} & \cdots & {\hat{\alpha }}_{1I}\\ \vdots & \ddots & \vdots & \ddots & \vdots \\ {\hat{\alpha }}_{i1} & \cdots & {\hat{\alpha }}_{ih} & \cdots & {\hat{\alpha }}_{iI}\\ \vdots & \ddots & \vdots & \ddots & \vdots \\ {\hat{\alpha }}_{I1} & \cdots & {\hat{\alpha }}_{Ih} & \cdots & {\hat{\alpha }}_{II} \end{array} & \begin{array}{ccccc} 0 & \cdots & 0 & \cdots & 0\\ \vdots & \ddots & \vdots & \ddots & \vdots \\ 0 & \cdots & 0 & \cdots & 0\\ \vdots & \ddots & \vdots & \ddots & \vdots \\ 0 & \cdots & 0 & \cdots & 0 \end{array} & \begin{array}{ccccc} 0 & \cdots & 0 & \cdots & 0\\ \vdots & \ddots & \vdots & \ddots & \vdots \\ 0 & \cdots & 0 & \cdots & 0\\ \vdots & \ddots & \vdots & \ddots & \vdots \\ 0 & \cdots & 0 & \cdots & 0 \end{array}\\ \begin{array}{ccccc} {\hat{\alpha }}_{11} & \cdots & {\hat{\alpha }}_{1r} & \cdots & {\hat{\alpha }}_{1R}\\ \vdots & \ddots & \vdots & \ddots & \vdots \\ {\hat{\alpha }}_{j1} & \cdots & {\hat{\alpha }}_{jr} & \cdots & {\hat{\alpha }}_{jR}\\ \vdots & \ddots & \vdots & \ddots & \vdots \\ {\hat{\alpha }}_{J1} & \cdots & {\hat{\alpha }}_{Jr} & \cdots & {\hat{\alpha }}_{JR} \end{array} & \begin{array}{ccccc} {\hat{b}}_{11} & \cdots & {\hat{b}}_{1s} & \cdots & {\hat{b}}_{1S}\\ \vdots & \ddots & \vdots & \ddots & \vdots \\ {\hat{b}}_{j1} & \cdots & {\hat{b}}_{js} & \cdots & {\hat{b}}_{jS}\\ \vdots & \ddots & \vdots & \ddots & \vdots \\ {\hat{b}}_{J1} & \cdots & {\hat{b}}_{Js} & \cdots & {\hat{b}}_{JS} \end{array} & \begin{array}{ccccc} -{\hat{c}}_{11} & \cdots & -{\hat{c}}_{1u} & \cdots & -{\hat{c}}_{1U}\\ \vdots & \ddots & \vdots & \ddots & \vdots \\ -{\hat{c}}_{j1} & \cdots & -{\hat{c}}_{ju} & \cdots & -{\hat{c}}_{jU}\\ \vdots & \ddots & \vdots & \ddots & \vdots \\ -{\hat{c}}_{J1} & \cdots & -{\hat{c}}_{Ju} & \cdots & -{\hat{c}}_{JU} \end{array}\\ \begin{array}{ccccc} {\hat{d}}_{11} & \cdots & {\hat{d}}_{1r} & \cdots & {\hat{d}}_{1R}\\ \vdots & \ddots & \vdots & \ddots & \vdots \\ {\hat{d}}_{x1} & \cdots & {\hat{d}}_{xr} & \cdots & {\hat{d}}_{xR}\\ \vdots & \ddots & \vdots & \ddots & \vdots \\ {\hat{d}}_{X1} & \cdots & {\hat{d}}_{Xr} & \cdots & {\hat{d}}_{XR} \end{array} & \begin{array}{ccccc} {\hat{f}}_{11} & \cdots & {\hat{f}}_{1s} & \cdots & {\hat{f}}_{1S}\\ \vdots & \ddots & \vdots & \ddots & \vdots \\ {\hat{f}}_{x1} & \cdots & {\hat{f}}_{xs} & \cdots & {\hat{f}}_{xS}\\ \vdots & \ddots & \vdots & \ddots & \vdots \\ {\hat{f}}_{X1} & \cdots & {\hat{f}}_{Xs} & \cdots & {\hat{f}}_{XS} \end{array} & \begin{array}{ccccc} -{\hat{q}}_{11} & \cdots & -{\hat{q}}_{1u} & \cdots & -{\hat{q}}_{1U}\\ \vdots & \ddots & \vdots & \ddots & \vdots \\ -{\hat{q}}_{x1} & \cdots & -{\hat{q}}_{xu} & \cdots & -{\hat{q}}_{xU}\\ \vdots & \ddots & \vdots & \ddots & \vdots \\ -{\hat{q}}_{X1} & \cdots & -{\hat{q}}_{Xu} & \cdots & -{\hat{q}}_{XU} \end{array}\\ \begin{array}{ccccc} {\hat{\eta }}_{11} & \cdots & {\hat{\eta }}_{1r} & \cdots & {\hat{\eta }}_{1R}\\ \vdots & \ddots & \vdots & \ddots & \vdots \\ {\hat{\eta }}_{y1} & \cdots & {\hat{\eta }}_{yr} & \cdots & {\hat{\eta }}_{yR}\\ \vdots & \ddots & \vdots & \ddots & \vdots \\ {\hat{\eta }}_{Y1} & \cdots & {\hat{\eta }}_{Yr} & \cdots & {\hat{\eta }}_{YR} \end{array} & \begin{array}{ccccc} {\hat{\mu }}_{11} & \cdots & {\hat{\mu }}_{1s} & \cdots & {\hat{\mu }}_{1S}\\ \vdots & \ddots & \vdots & \ddots & \vdots \\ {\hat{\mu }}_{y1} & \cdots & {\hat{\mu }}_{ys} & \cdots & {\hat{\mu }}_{yS}\\ \vdots & \ddots & \vdots & \ddots & \vdots \\ {\hat{\mu }}_{Y1} & \cdots & {\hat{\mu }}_{Ys} & \cdots & {\hat{\mu }}_{YS} \end{array} & \begin{array}{ccccc} -{\hat{\nu }}_{11} & \cdots & -{\hat{\nu }}_{1u} & \cdots & -{\hat{\nu }}_{1U}\\ \vdots & \ddots & \vdots & \ddots & \vdots \\ -{\hat{\nu }}_{y1} & \cdots & -{\hat{\nu }}_{yu} & \cdots & -{\hat{\nu }}_{yU}\\ \vdots & \ddots & \vdots & \ddots & \vdots \\ -{\hat{\nu }}_{Y1} & \cdots & -{\hat{\nu }}_{Yu} & \cdots & -{\hat{\nu }}_{YU} \end{array} \end{array}]\in {ℜ}^{\left(I^{*}+J^{*}+X^{*}+Y^{*})\times (R^{*}+S^{*}+U^{*}\right)}$$

where ${\hat{\alpha }}_{ih}$ is the estimated interactive ability between the *i*-th protein and the *h*-th protein; ${\hat{a}}_{jr}$, ${\hat{b}}_{js}$, and ${\hat{c}}_{ju}$ individually represent the estimated regulation abilities of the *r*-th TF on the *j*-th gene, the *s*-th lncRNA on the *j*-th gene, and the *u*-th miRNA on the *j*-th gene; ${\hat{d}}_{xr}$, ${\hat{f}}_{xs}$, and ${\hat{q}}_{xu}$ are separately the *r*-th TF on the *x*-th lncRNA, the *s*-th lncRNA on the *x*-th lncRNA, and the *u*-th miRNA on the *x*-th lncRNA; and ${\hat{\eta }}_{yr}$, ${\hat{\mu }}_{ys}$, and ${\hat{\nu }}_{yu}$ indicate the *r*-th TF on the *y*-th miRNA, the *s*-th lncRNA on the *y*-th miRNA, and the *u*-th miRNA on the *y*-th miRNA.

Subsequently, we performed singular value decomposition (SVD) on the system matrix *K*.

The SVD of *K* is described as follows:

(17) $$K=BDZ^{T}$$

where $B\in \;{ℜ}^{\left(I^{*}+J^{*}+X^{*}+Y^{*})\times (I^{*}+J^{*}+X^{*}+Y^{*}\right)}$ and $Z^{T}\in \;{ℜ}^{\left(R^{*}+S^{*}+U^{*})\times (R^{*}+S^{*}+U^{*}\right)}$ denote the unitary singular matrix and $D=diag(d_{1},\ldots ,d_{i},\ldots ,d_{R*+S*+U*})\in {ℜ}^{\left(I^{*}+J^{*}+X^{*}+Y^{*})\times (R^{*}+S^{*}+U^{*}\right)}$ indicates the diagonal matrix composed of $R^{*}+S^{*}+U^{*}$ singular values in descending order (i.e., $d_{1}\geq \cdots \geq d_{i}\geq \cdots \geq d_{R*+S*+U*}$). Then, we normalize the singular values as below:

(18) $$P_{i}=\frac{d_{i}^{2}}{\sum_{i=1}^{R*+S*+U*}d_{i}^{2}}$$

From the viewpoint of energy, the top *r* singular values represent 85% of the principal network (i.e., $\sum_{i=1}^{I}P_{i}\geq 0.85$). Subsequently, we project each node in the system matrix *K* to the *r*-th singular vector. The corresponding equation is:

(19) $$Z(t,r)=k_{t,:}\cdot z_{:,r}^{T},\;for\;t=1,\ldots I*+J*+X*+Y*,\;r=1,\ldots I$$

where $k_{t,:}$ denotes the *t*-th row vector of *K* and $z_{:,r}^{T}$ is the *r*-th singular vector. We define the 2-norm projection value for each node, including protein, gene, lncRNA, and miRNA, on the 85% principal structure of the real GWGEN as below:

(20) $$R(t)=\sqrt{\sum_{r=1}^{I}Z^{2}(t,r)},\;\mathrm{for}\;t=1,\ldots I*+J*+X*+Y*,\;r=1,\;\ldots ,\;I$$

If the value of R(t) is larger, the *t*-th node is more important to the principal structure. On the other hand, if the projection value of R(t) is close to zero, the relevant node is insignificant and practically independent of the principal structure of the real GWGEN. According to the projection value, we chose the top 3000 nodes to construct the core GWGENs of normal prostate cells (including lean and obese groups), and lean and obese PCa, as shown in Figures S5–S8. Combining this with the literature survey, we can obtain core signaling pathways in the annotation of KEGG pathways. The core signaling pathways of normal prostate cells (including lean and obese groups), and lean and obese PCa refer to Figures S9–S12, respectively.

### 4.6. Deep-Neural-Network-Based Drug–Target Interaction Prediction Model

Investigating the core signaling pathways, we identified essential biomarkers based on carcinogenic molecular mechanisms for PCa (covering lean and obese groups) and obesity-specific PCa. The corresponding biomarkers (drug-targets) are shown in Table 1. The systems drug discovery method was proposed to design multiple-molecule drugs targeting these biomarkers. With the help of the DNN-based DTI model, we considered the drug–target interaction probability, which was one of the drug design specifications.

As shown in the flowchart for system drug discovery and design in Figure 3, the drug–target interaction dataset was from BindingDB [61]. In order to delineate the drug–target interactions in a numerical vector, we transformed them into a feature vector using the PyBioMed Python package in a Python 2.7 environment [84]. We used the PyMolecule module in PyBioMed to transform the drug descriptors. The drug features contain commonly used structural and physicochemical information. The PyProtein module in PyBioMed was applied to transform the target descriptors. The target features were computed based on the widely used structural and physicochemical properties of proteins and peptides from amino acid sequences. The total numbers of drug and target features were 363 and 996, respectively. More details about descriptor transformation are given in the PyBioMed documentation. In this way, we could use a united vector to describe a drug–target interaction pair, as shown below:

(21) $$\upsilon_{drug-t\arg et}=[W,\;F]=[w_{1},w_{2},\ldots ,w_{x},f_{1},f_{2},\ldots ,f_{y}]\;$$

where $\upsilon_{drug-t\arg et}$ denotes the feature vector of a drug–target pair; *x* and *y* are the total number of drug features and target features, respectively; W and F individually represent the feature vector of the relevant drug and relevant target; $w_{x}$ is the *x*-th drug feature; and $f_{y}$ is the *y*-th target feature. Moreover, before training the DNN-based DTI model, we scaled the data via standardization, since the drug and target features are measured on different scales. Lastly, to remove noisy features and reduce memory consumption, we applied principal component analysis (PCA), to decrease the feature size from 1359 to 618 [85].

For the DNN-based DTI model, each output layer could be described as follows:

(22) $$k_{n}=\delta (w^{T}x_{n}+b),\;\mathrm{where}\;w=\left[\begin{array}{c} w_{1}\\ w_{2}\\ \vdots \\ w_{h} \end{array}\right],\;b=\left[\begin{array}{c} b_{1}\\ b_{2}\\ \vdots \\ b_{h} \end{array}\right]$$

where $k_{n}$ represents the output of each layer when input vector $x_{n}$ is the *n*-th drug–target vector; δ denotes the activation function (ReLU for the hidden layers and sigmoid for the output layer); w represents the weighting parameters; and b signifies bias parameters. The drug–target interaction prediction is a binary classification problem. Hence, the binary cross-entropy is selected to be the cost function:

(23) $$\begin{array}{l} C_{n}(w,b)=-\frac{1}{N}\sum_{n=1}^{N}(s_{n}\log({\hat{s}}_{n})+(1-s_{n})\log(1-{\hat{s}}_{n}))\\ L(w,b)=\frac{1}{N}\sum_{n=1}^{N}C_{n}(s_{n},{\hat{s}}_{n}) \end{array}$$

where L(w,b) denotes the average total loss; $s_{n}$ is the n-th true positive instance (1) or true negative instance (0) of drug–target binding; and ${\hat{s}}_{n}$ denotes the *n*-th predicted probability of a positive instance (1) or predicted probability of a negative instance (0) of drug–target binding. For obtaining the optimal network parameter set $\phi^{*}$, the cost function is used in the following:

(24) $$\phi^{*}=\arg\underset{\phi }{\min}\;L(\phi )$$

The above equation could be achieved using the backpropagation algorithm [86]. The updated weight and bias parameters for the *j*-th epoch are shown below:

(25) $$\begin{array}{l} \phi^{j}=\phi^{j-1}-\eta \nabla L(\phi^{j-1}),\\ \mathrm{where}\;\nabla L(\phi^{j-1})=\left[\begin{array}{c} \frac{\partial L(\phi^{j-1})}{\partial w_{1}}\\ \vdots \\ \frac{\partial L(\phi^{j-1})}{\partial w_{h}}\\ \frac{\partial L(\phi^{j-1})}{\partial b_{1}}\\ \vdots \\ \frac{\partial L(\phi^{j-1})}{\partial b_{h}} \end{array}\right]. \end{array}$$

where η is the learning rate, which is 0.001 and $\nabla L(\phi^{j-1})$ denotes the gradient of $L(\phi^{j-1})$.

## 5. Conclusions

In this study, we proposed a systems biology approach to investigating the role of obesity in PCa and identifying essential biomarkers as drug targets for PCa (covering lean and obese) and obesity-specific PCa. In addition, we provided drug design specifications, including drug–target interaction, drug regulation ability, and drug toxicity. For considering the drug–target interactions, we trained a DNN-based DTI model in advance. Utilizing this, we could obtain predicted drug candidates based on the identified biomarkers. These drug candidates then passed through filters for drug regulation ability and drug toxicity. Finally, we suggested two potential multiple-molecule drugs (drug combinations) to prevent PCa (covering lean and obese) and obesity-specific PCa. Nonetheless, there is still room for improvement, especially in the case of leveraging more genomics data and applying advanced DNN-based DTI models that consider the compounds’ chemical structures in graphs to enhance the pipeline. Moreover, it is noted that the process of developing a novel drug is time-consuming, risky, and costly. By combining the proposed systems biology approach with computational drug discovery, the steps of target identification and target validation in drug discovery might be accelerated and optimized before the drug enters clinical trials.

## Figures and Tables

**Table 1:** The identified biomarkers (drug targets) for PCa and obesity-specific PCa.

Disease	Drug Targets
PCa (covering lean and obese)	STAT1, *FOXF2*, SIM2, SMAD2, MYB, *EGFR*
Obesity-specific PCa	STAT1, *FOXF2*, SIM2, SMAD2, CERK, STAT3, TP53

**Table 2:** Potential multiple-molecule drug and the corresponding target genes for PCa.

	Targets	STAT1	*FOXF2*	SIM2	SMAD2	MYB	*EGFR*
Drugs							
Apigenin		▪		▪		▪	▪
Digoxin			▪		▪		

**Table 3:** Potential multiple-molecule drug and the corresponding target genes for obese PCa.

	Targets	STAT1	*FOXF2*	SIM2	SMAD2	CERK	STAT3	TP53
Drugs								
Apigenin		▪		▪		▪	▪	
Digoxin			▪		▪	▪		▪
Orlistat			▪	▪		▪		▪

## Supplementary Materials

The following supporting information can be downloaded online. Figure S1: The real genome-wide genetic and epigenetic network (GWGEN) of normal prostate cells in the lean group, Figure S2: The real genome-wide genetic and epigenetic network (GWGEN) of normal prostate cells in the obese group, Figure S3: The real genome-wide genetic and epigenetic network (GWGEN) of lean PCa, Figure S4: The real genome-wide genetic and epigenetic network (GWGEN) of obese PCa, Figure S5: The core genome-wide genetic and epigenetic network (GWGEN) of normal prostate cells in the lean group, Figure S6: The core genome-wide genetic and epigenetic network (GWGEN) of normal prostate cells in the obese group, Figure S7: The core genome-wide genetic and epigenetic network (GWGEN) of lean PCa, Figure S8: The core genome-wide genetic and epigenetic network (GWGEN) of obese PCa, Figure S9: The core signaling pathways to investigate the healthy mechanism of normal prostate cells in the lean group, Figure S10: The core signaling pathways to investigate the healthy mechanism of normal prostate cells in the obese group, Figure S11: The core signaling pathways to investigate the carcinogenic mechanism of lean PCa, Figure S12: The core signaling pathways to investigate the carcinogenic mechanism of obese PCa, Figure S13: The core signaling pathways integrated from core signaling pathways of normal prostate cells (lean group) in Figure S9 and lean PCa in Figure S11, Figure S14: The core signaling pathways integrated from core signaling pathways of normal prostate cells (obese group) in Figure S10 and obese PCa in Figure S12, Figure S15: The structure of DTI model, Figure S16: The accuracy and loss for training and validation sets by 10-fold cross validation, Figure S17: The ROC curves of different models for the drug-target interaction prediction, Table S1: The overall statistical table of nodes and edges in the candidate GWGEN and real GWGENs of normal prostate cells (including lean and obese groups), lean, and obese PCa after system identification, Table S2: Enrichment analysis in core GWGEN of normal prostate cells (lean group) by the DAVID, Table S3: Enrichment analysis in core GWGEN of normal prostate cells (obese group) by the DAVID, Table S4: Enrichment analysis in core GWGEN of lean PCa by the DAVID, Table S5: Enrichment analysis in core GWGEN of obese PCa by the DAVID, Table S6: Model performance of DNN-based DTI model (10-fold cross validation), Table S7: The candidate drugs identified for target STAT1, *FOXF2*, SIM2, SMAD2, MYB, *EGFR*, CERK, STAT3 and TP53.

## Sample Availability

Samples of the compounds are not available from the authors.
