# Supplementary material for: Investigating the Role of Obesity in Prostate Cancer and Identifying Biomarkers for Drug Discovery: Systems Biology and Deep Learning Approaches
Source: Molecules. 2022 Jan 28;27(3):900. doi: 10.3390/molecules27030900 (PMC8840188; doi:10.3390/molecules27030900)
Supplement: Supplementary file 1 [file molecules-27-00900-s001.zip › molecules-1559000-supplementary.pdf]

1.1 System modeling for normal prostate cells and PCa

The x-th lncRNA is influenced by TFs, lncRNAs, and miRNAs. Hence, the x-th lncRNA in the candidate lncRNA regulatory network (LRN) model can be written in the following regulatory equation:

$$l_x[n] = \sum_{r=1}^{R_x} d_{xr} T_r[n] + \sum_{\substack{s=1 \\ s\neq x}}^{S_x} f_{xs} L_s[n] - \sum_{u=1}^{U_x} q_{xu} M_u[n] l_x[n] + \lambda_x + \phi_x[n], \quad \text{for } x = 1,\dots,X,\ n = 1,\dots,N \tag{S1}$$

where $l_x[n]$ refers to the expression level of the x-th lncRNA; R_x, S_x, and U_x indicate total number of TFs binding to the x-th lncRNA, the total number of lncRNAs binding to the x-th lncRNA, and the total number of miRNAs impeding the x-th lncRNA respectively. $T_r[n]$, $L_s[n]$, and $M_u[n]$ individually denote the expression level of the r-th TF, the s-th lncRNA, and the u-th miRNA for the n-th sample; d_{xr} and f_{xs} respectively represent the transcriptional regulatory ability from the r-th TF and the s-th lncRNA to the x-th lncRNA; $-q_{xu} \leq 0$ demonstrates the post-transcriptional regulatory ability from the u-th miRNA to the x-th lncRNA; N and X respectively represent the total number of samples and lncRNAs; λ_x is the basal level of the x-th lncRNA owing to unknown regulations; $\phi_x[n]$ denotes the stochastic noise.

In the same way, the expression of the y-th miRNA is influenced by the TFs, lncRNAs, miRNAs as well. In addition, the y-th miRNA in the candidate miRNA regulatory network (MRN) model among candidate GWGENs can be explained by the following regulatory equations:

$$m_y[n] = \sum_{r=1}^{R_y} \eta_{yr} T_r[n] + \sum_{s=1}^{S_y} \mu_{ys} L_s[n] - \sum_{u=1}^{U_y} v_{yu} M_u[n] m_y[n] + \lambda_y + \phi_y[n], \quad \text{for } y = 1,\dots,Y,\ n = 1,\dots,N \tag{S2}$$

where $m_y[n]$ denotes the expression level of the y-th miRNA; η_{yr} and μ_{ys} respectively represent the transcription regulatory ability from the r-th TF and the s-th lncRNA to the y-th miRNA; $-v_{yu} \leq 0$ indicates the post-transcription regulatory ability by which the u-th miRNA inhibits the y-th miRNA; $T_r[n]$, $L_s[n]$, and $M_u[n]$ are individually the expression of the r-th TF, the s-th lncRNA, and the y-th miRNA; R_y denotes the total number of TFs binding to the y-th miRNA; S_y denotes the total

number of lncRNAs binding to the y-th miRNA; U_y represents the total number of miRNAs restraining the y-th miRNA. N and Y are respectively the total number of data samples and miRNAs; λ_y denotes the basal level of the y-th miRNA expression due to unknown regulations; $\phi_y[n]$ represents the stochastic noise of gene expression in the y-th miRNA for the sample n.

1.2 Utilizing system identification and system order detection methods to identify real GWGENs from the candidate GWGEN

After formulating lncRNA and miRNA in the candidate GWGEN by equations (S1) and (S2), we are going to estimate regulation parameters with the help of microarray data. Hence, firstly, we can rewrite equations (S1) and (S2) in the following equations:

$$l_x[n] = \left[T_1[n] \cdots T_{R_x}[n] \;\; L_1[n] \cdots L_{S_x}[n] \;\; g_x[n]M_1[n] \cdots g_x[n]M_{U_x}[n] \;\; 1 \right] \times \begin{bmatrix} d_{x1} \\ \vdots \\ d_{xR_x} \\ f_{x1} \\ \vdots \\ f_{xS_x} \\ -q_{x1} \\ \vdots \\ -q_{xU_x} \\ \lambda_x \end{bmatrix} + \phi_x[n] \qquad \text{(S3)}$$

$$m_y[n] = \left[T_1[n] \cdots T_{R_y} \;\; L_1[n] \cdots L_{S_y}[n] \;\; g_y[n]M_1[n] \cdots g_y[n]M_{U_y}[n] \;\; 1 \right] \times \begin{bmatrix} \eta_{y1} \\ \vdots \\ \eta_{yR_y} \\ \mu_{y1} \\ \vdots \\ \mu_{yS_y} \\ -v_{y1} \\ \vdots \\ -v_{yU_y} \\ \lambda_y \end{bmatrix} + \phi_y[n] \qquad \text{(S4)}$$

For simplicity, the above equations can be represented by the following equations:

$$l_x[n] = \beta_{x,L}[n] \cdot \gamma_{x,L} + \phi_x[n], \quad \text{for } x = 1,\ldots,X \quad n = 1,\ldots,N \qquad \text{(S5)}$$

$$m_y[n] = \beta_{y,M}[n] \cdot \gamma_{y,M} + \phi_y[n],\ \text{for}\, y = 1,\ldots,Y\ \ n = 1,\ldots,N \tag{S6}$$

Where $\gamma_{x,L}$ and $\gamma_{y,M}$ individually represent the estimated parameters of post-transcriptional regulation abilities from lncRNA and miRNA; $\beta_{x,L}[n]$ and $\beta_{y,M}[n]$ separately denote expression vector of lncRNA and miRNA for the sample n.

Since we have N samples, the equations (S5) and (S6) could be augmented in the following forms:

$$\begin{bmatrix} l_x[1] \\ l_x[2] \\ \vdots \\ l_x[N] \end{bmatrix} = \begin{bmatrix} \beta_{x,L}[1] \\ \beta_{x,L}[2] \\ \vdots \\ \beta_{x,L}[N] \end{bmatrix} \cdot \gamma_{x,L} + \begin{bmatrix} \phi_x[1] \\ \phi_x[2] \\ \vdots \\ \phi_x[N] \end{bmatrix} \tag{S7}$$

$$\begin{bmatrix} m_y[1] \\ m_y[2] \\ \vdots \\ m_y[N] \end{bmatrix} = \begin{bmatrix} \beta_{y,M}[1] \\ \beta_{y,M}[2] \\ \vdots \\ \beta_{y,M}[N] \end{bmatrix} \cdot \gamma_{y,M} + \begin{bmatrix} \phi_y[1] \\ \phi_y[2] \\ \vdots \\ \phi_y[N] \end{bmatrix} \tag{S8}$$

Additionally, (S7) and (S8) are equal to the equations as below:

$$L_x = \varepsilon_{x,L} \cdot \gamma_{x,L} + \varphi_x \tag{S9}$$

$$M_y = \varepsilon_{y,M} \cdot \gamma_{y,M} + \varphi_y \tag{S10}$$

In order to obtain the estimated parameters in equations (S9) and (S10), we are going to solve the constrained linear least squares estimation problems in the following:

$$\hat{\gamma}_{x,L} = \min_{\gamma_x, L} \frac{1}{2} \left\| \varepsilon_{x,L} \cdot \gamma_{x,L} - L_x \right\|_2^2 \tag{S11}$$

$$
\text{subject to} \begin{bmatrix} 0 & \cdots & \cdots & 0 & 0 & \cdots & \cdots & 0 & 1 & 0 & \cdots & 0 & 0 \\ \vdots & \ddots & & \vdots & \vdots & \ddots & & \vdots & 0 & \ddots & \ddots & \vdots & \vdots \\ \vdots & & \ddots & \vdots & \vdots & & \ddots & \vdots & \vdots & \ddots & \ddots & 0 & \vdots \\ 0 & \cdots & \cdots & 0 & 0 & \cdots & \cdots & 0 & 0 & \cdots & 0 & 1 & 0 \end{bmatrix} \gamma_{x,L} \le \begin{bmatrix} 0 \\ \vdots \\ \vdots \\ 0 \end{bmatrix}
$$

$$
\qquad\qquad R_x \qquad\qquad\qquad S_x \qquad\qquad\qquad U_x
$$

$$
\hat{\gamma}_{y,M} = \min_{\gamma_y,M} \frac{1}{2} \left\| \varepsilon_{y,M} \cdot \gamma_{y,M} - M_y \right\|_2^2 \tag{S12}
$$

$$
\text{subject to} \begin{bmatrix} 0 & \cdots & \cdots & 0 & 0 & \cdots & \cdots & 0 & 1 & 0 & \cdots & 0 & 0 \\ \vdots & \ddots & & \vdots & \vdots & \ddots & & \vdots & 0 & \ddots & \ddots & \vdots & \vdots \\ \vdots & & \ddots & \vdots & \vdots & & \ddots & \vdots & \vdots & \ddots & \ddots & 0 & \vdots \\ 0 & \cdots & \cdots & 0 & 0 & \cdots & \cdots & 0 & 0 & \cdots & 0 & 1 & 0 \end{bmatrix} \gamma_{y,M} \le \begin{bmatrix} 0 \\ \vdots \\ \vdots \\ 0 \end{bmatrix}
$$

$$
\qquad\qquad R_y \qquad\qquad\qquad S_y \qquad\qquad\qquad U_y
$$

It is noted that the matrix inequality in the equations (S11) and (S12) could guarantee that the estimated post-transcriptional regulatory abilities of miRNA are negative. Further, the constrained linear least squares estimation problem could be solved via MATLAB optimization toolbox.

Building the candidate GWGEN from various database, there may exist false-positive interactions. Therefore, we are going to do system order detection by computing the AIC. Based on the AIC theory, the real system would lead to the smallest AIC value. The AIC of the x-th lncRNA and the y-th miRNA are given as below:

$$
\begin{aligned}
&AIC(R_x, S_x, U_x) = \log(\hat{\hbar}_{x,L}^2) + \frac{2(\bar{\lambda}_{x,L})}{N} \\
&\text{where } \hat{\hbar}_{x,L} = \sqrt{\frac{(L_x - (\varepsilon_{x,L} \cdot \hat{\gamma}_{x,L}))^T (L_x - (\varepsilon_{x,L} \cdot \hat{\gamma}_{x,L}))}{N}}, \bar{\lambda}_{x,L} = R_x + S_x + U_x + 1
\end{aligned} \tag{S13}
$$

$$
\begin{aligned}
&AIC(R_y, S_y, U_y) = \log(\hat{\hbar}_{y,M}^2) + \frac{2(\bar{\lambda}_{y,M})}{N} \\
&\text{where } \hat{\hbar}_{y,M} = \sqrt{\frac{(M_y - (\varepsilon_{y,M} \cdot \hat{\gamma}_{y,M}))^T (M_y - (\varepsilon_{y,M} \cdot \hat{\gamma}_{y,M}))}{N}}, \bar{\lambda}_{y,M} = R_y + S_y + U_y + 1
\end{aligned} \tag{S14}
$$

where $\hat{\hbar}_{x,L}^2$ and $\hat{\hbar}_{y,M}^2$ individually denote the estimated residual error of the x-th lncRNA and the y-th miRNA; $\bar{\lambda}_{x,L}$ and $\bar{\lambda}_{y,M}$ are the number (order) of the parameters of the x-th lncRNA in the parameter estimation problem in (s11) and the number (order) of parameters of the y-th lncRNA in the parameter estimation problem in (S12). Moreover, the estimated regulation abilities $\hat{\gamma}_{x,L}$ and $\hat{\gamma}_{y,M}$ can be obtained after

solving optimization problem in (S11) and (S12). The real system order, which are R_x^*, S_x^*, U_x^* for the x-th lncRNA and R_y^*, S_y^*, U_y^* for the y-th miRNA, can lead to the smallest AIC in (S13) and (S14), respectively. In other words, the insignificant interactions and regulations out of the real system order will be removed.

Tables

Table S1. The overall statistical table of nodes and edges in the candidate GWGEN and real GWGENs of normal prostate cells (including lean and obese groups), lean, and obese PCa after system identification.

Node/edge	candidate	normal(lean)	normal(obese)	lean PCa	obese PCa
LncRNA-TF	59	1	1	1	1
LncRNA-Receptor	2	1	2	2	1
LncRNA-Protein	49	13	9	10	8
LncRNA	151	126	103	107	106
MiRNA- LncRNA	245	1	1	2	3
MiRNA- MiRNA	6	6	6	6	5
MiRNA-TF	16338	21	30	25	20
MiRNA-Receptor	13638	43	35	34	43
MiRNA-Protein	74055	211	186	173	189
MiRNA	197	134	139	144	138
TF- LncRNA	210	49	60	62	69
TF-MiRNA	1422	118	139	120	124
TF- TF	31274	231	204	223	199
TF- Receptor	17071	1227	1062	1182	1223
TF- Protein	86688	6695	5961	6229	6585
TF	2049	180	207	210	217
Receptor- LncRNA	30	90	29	28	83
Receptor- MiRNA	147	128	124	138	146
Receptor- TF	2380	288	270	281	278
Receptor- Receptor	1756	1636	1527	1674	1715
Receptor- Protein	8887	7972	8247	8477	8275
Receptor	2129	2029	2113	2087	2087
Protein	14218	13255	11995	11874	11874
PPI edge	4237685	857190	840614	854637	856258
Total node	18744	15724	14557	14422	14422
Total edge	4491942	868695	858558	873377	876234

Table S2. Enrichment analysis in core GWGEN of normal prostate cells (lean group) by the DAVID.

Term	Numbers	p-value
Adipocytokine signaling pathway	21	4.3×10^{-3}
Taste transduction	15	7.1×10^{-3}
Cell cycle	32	4.5×10^{-3}
HIF-1 signaling pathway	25	1.2×10^{-2}
PI3K-Akt signaling pathway	66	6.0×10^{-2}

Table S3. Enrichment analysis in core GWGEN of normal prostate cells (obese group) by the DAVID.

Term	Numbers	p-value
Oxidative phosphorylation	31	4.5×10^{-3}
Protein digestion and absorption	22	8.3×10^{-3}
cAMP signaling pathway	38	3.6×10^{-2}
HIF-1 signaling pathway	21	4.0×10^{-2}
Glutathione metabolism	13	4.5×10^{-2}

Table S4. Enrichment analysis in core GWGEN of lean PCa by the DAVID.

Term	Numbers	p-value
Pathways in cancer	71	5.0×10^{-2}
T-cell receptor signaling pathway	21	9.4×10^{-2}
MAPK signaling pathway	49	3.1×10^{-2}
mTOR signaling pathway	14	7.2×10^{-2}
Prostate cancer	19	8.3×10^{-2}

Table S5. Enrichment analysis in core GWGEN of obese PCa by the DAVID.

Term	Numbers	p-value
Pathways in cancer	67	9.4×10^{-2}
PI3K-Akt signaling pathway	60	8.4×10^{-2}
MAPK signaling pathway	46	7.3×10^{-2}
mTOR signaling pathway	14	6.5×10^{-2}
Prostate cancer	19	8.3×10^{-2}

Table S6. Model performance of DNN-based DTI model (10-fold cross validation).

	Validation loss	Validation accuracy (%)	Testing loss	Testing accuracy (%)
**1**	0.148	95.23	0.159	94.87
**2**	0.151	94.93	0.150	95.05
**3**	0.159	94.58	0.155	94.69
**4**	0.155	94.73	0.161	94.68
**5**	0.154	94.75	0.156	94.91
**6**	0.147	94.91	0.155	94.95
**7**	0.164	94.74	0.157	94.96
**8**	0.162	94.56	0.158	94.82
**9**	**0.151**	**95.18**	**0.155**	**95.06**
**10**	0.142	95.20	0.153	94.94
**Average**	0.153	94.88	0.156	94.89
**Standard deviation**	0.007	0.252	0.003	0.131

Table S7. The candidate drugs identified for target STAT1, *FOXF2*, SIM2, SMAD2, MYB, *EGFR*, CERK, STAT3 and TP53.

**STAT1**			***FOXF2***		
**Drug**	**Toxicity (LD50, mol/kg)**	**Regulation ability (CMap)**	**Drug**	**Toxicity (LD50, mol/kg)**	**Regulation ability (CMap)**
Prochlorperazine	2.3496	-0.16563	Gliclazide	2.0016	0.02484
Rosiglitazone	2.4515	-0.15607	Prilocaine	2.1374	0.00225
Apigenin	2.6983	-0.22592	Orlistat	2.3363	0.46585
Triflupromazine	3.2485	-0.17981	Bromocriptine	2.7499	0.07726
Methotrexate	3.4955	-0.20734	Digoxin	4.4721	0.27600
**SIM2**			**SMAD2**		
**Drug**	**Toxicity (LD50, mol/kg)**	**Regulation ability (CMap)**	**Drug**	**Toxicity (LD50, mol/kg)**	**Regulation ability (CMap)**
Gliclazide	2.0016	-0.09183	Omeprazole	2.2254	0.15675
Orlistat	2.3363	-0.00007	Cefotiam	2.4511	0.23653
Apigenin	2.6983	-0.15210	Thiethylperazine	2.5624	0.13800
Bromocriptine	2.7499	-0.22652	Alfuzosin	2.6826	0.17104

Tiratricol	3.8459	-0.12997	Digoxin	4.4721	0.25736

**MYB**			***EGFR***		
**Drug**	**Toxicity (LD50, mol/kg)**	**Regulation ability (CMap)**	**Drug**	**Toxicity (LD50, mol/kg)**	**Regulation ability (CMap)**
Rosiglitazone	2.4515	-0.05222	Riboflavin	1.6067	-0.11210
Apigenin	2.6983	-0.19442	Gliclazide	2.0016	-0.17868
Fluphenazine	2.8990	-0.70223	Betaxolol	2.1620	-0.07627
Perphenazine	3.0725	-0.13066	Apigenin	2.6983	-0.32192
Chlorpromazine	3.3196	-0.20530	Fluvoxamine	2.6997	-0.07871

**CERK**			**STAT3**		
**Drug**	**Toxicity (LD50, mol/kg)**	**Regulation ability (CMap)**	**Drug**	**Toxicity (LD50, mol/kg)**	**Regulation ability (CMap)**
Orlistat	2.3363	-0.00030	Fenoprofen	1.9985	-0.19561
Apigenin	2.6983	-0.15082	Nizatidine	2.4350	-0.15898
Bromocriptine	2.7499	-0.10850	Tridihexethyl	2.5261	-0.01752
Indometacin	4.0722	-1.00000	Apigenin	2.6983	-0.23711
Digoxin	4.4721	-0.00055	Lisuride	3.1801	-0.09357

**TP53**		
**Drug**	**Toxicity (LD50, mol/kg)**	**Regulation ability (CMap)**
Orciprenalin	1.8283	0.03048
Prilocaine	2.1374	0.03838
Orlistat	2.3363	0.46450
Bromocriptine	2.7499	0.05684
Digoxin	4.4721	0.21932

Figures

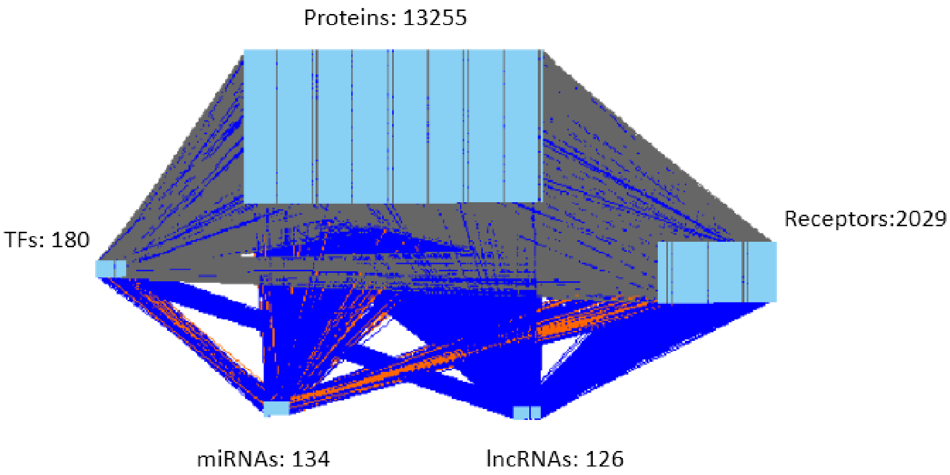

Figure S1. The real genome-wide genetic and epigenetic network (GWGEN) of normal prostate cells in the lean group. The lines in color of grey represent protein-protein interactions (PPIs); The lines in color of blue indicate transcriptional regulations by TFs and lncRNAs; The lines in color of orange refer to post-transcriptional regulations by miRNAs; The numbers of Proteins, Receptors, TFs, miRNAs and lncRNAs are 13255, 2029, 180, 134 and 126, respectively.

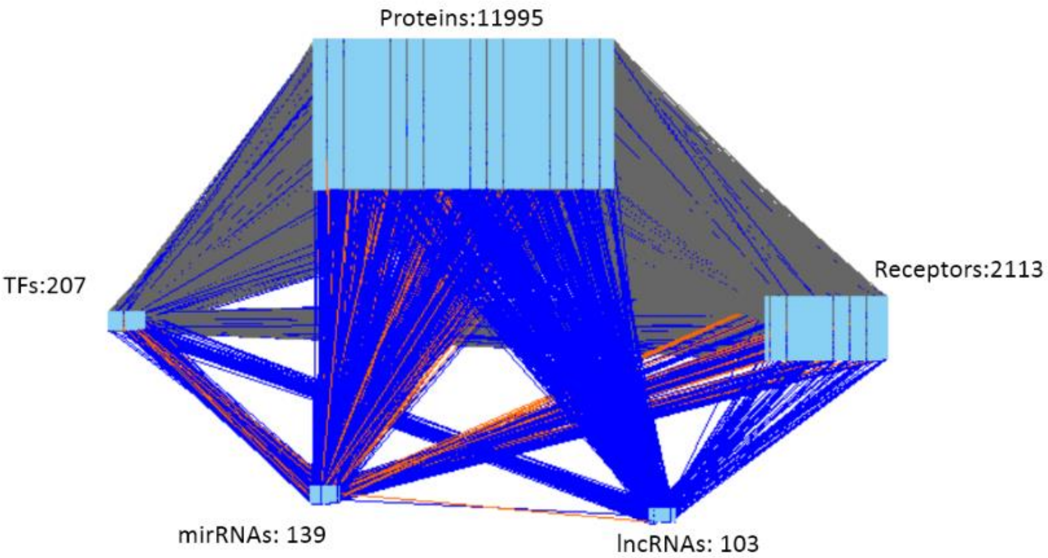

Figure S2. The real genome-wide genetic and epigenetic network (GWGEN) of normal prostate cells in the obese group. The numbers of Proteins, Receptors, TFs, miRNAs and lncRNAs are 11995, 2113, 207, 139 and 103, respectively.

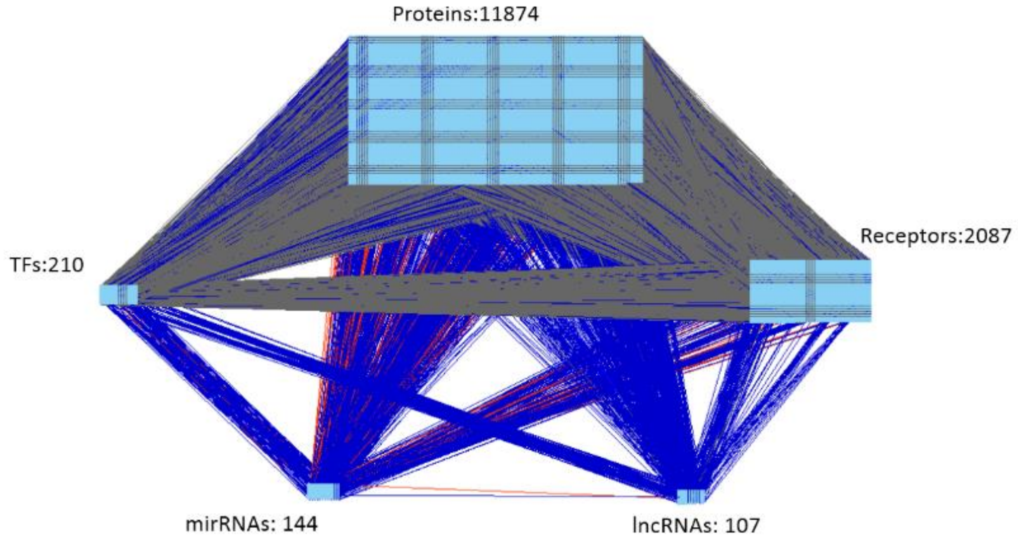

Figure S3. The real genome-wide genetic and epigenetic network (GWGEN) of lean PCa. The numbers of Proteins, Receptors, TFs, miRNAs and lncRNAs are 11874, 2087, 210, 144 and 107, respectively.

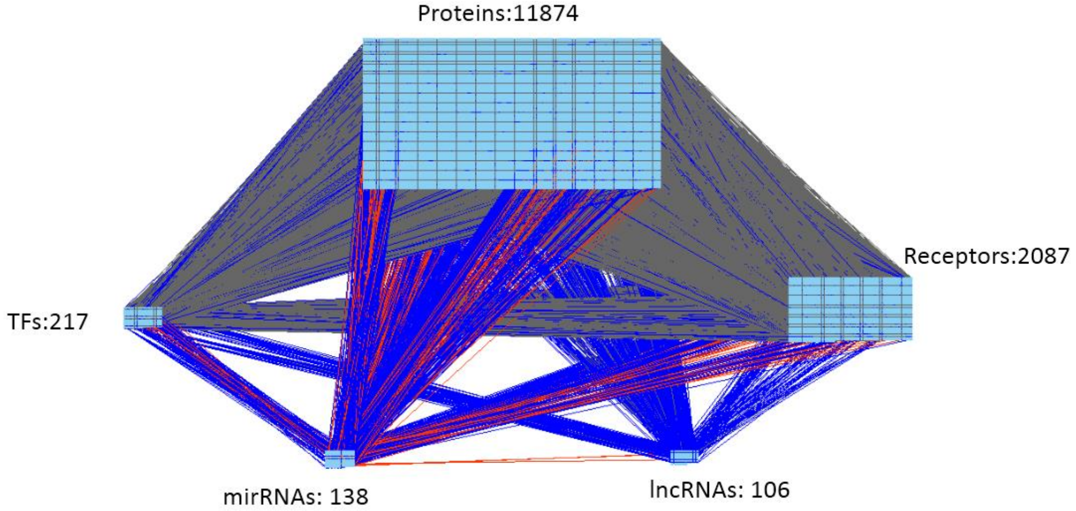

Figure S4. The real genome-wide genetic and epigenetic network (GWGEN) of obese PCa. The numbers of Proteins, Receptors, TFs, miRNAs and lncRNAs are 11874, 2087, 217, 138 and 106, respectively.

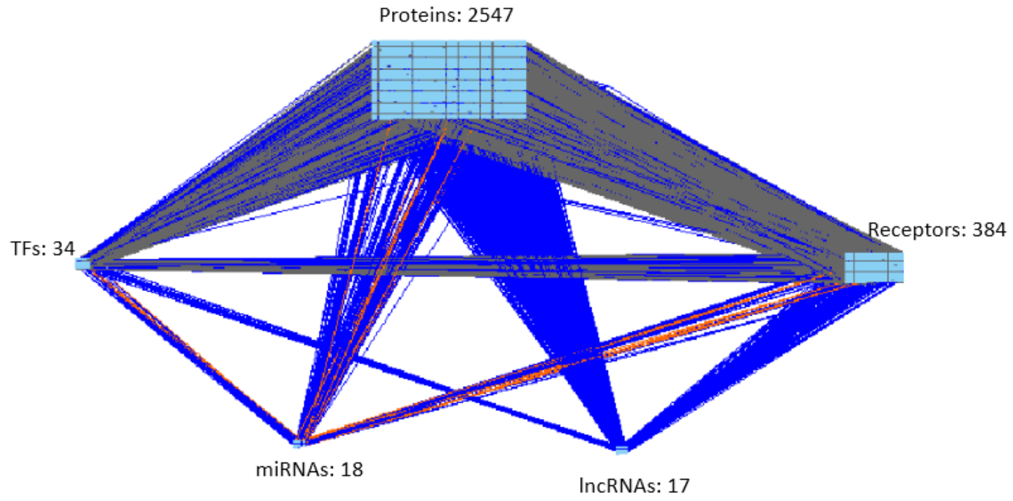

Figure S5. The core genome-wide genetic and epigenetic network (GWGEN) of normal prostate cells in the lean group. The numbers of Proteins, Receptors, TFs, miRNAs and lncRNAs are 2547, 384, 34, 18 and 17, respectively.

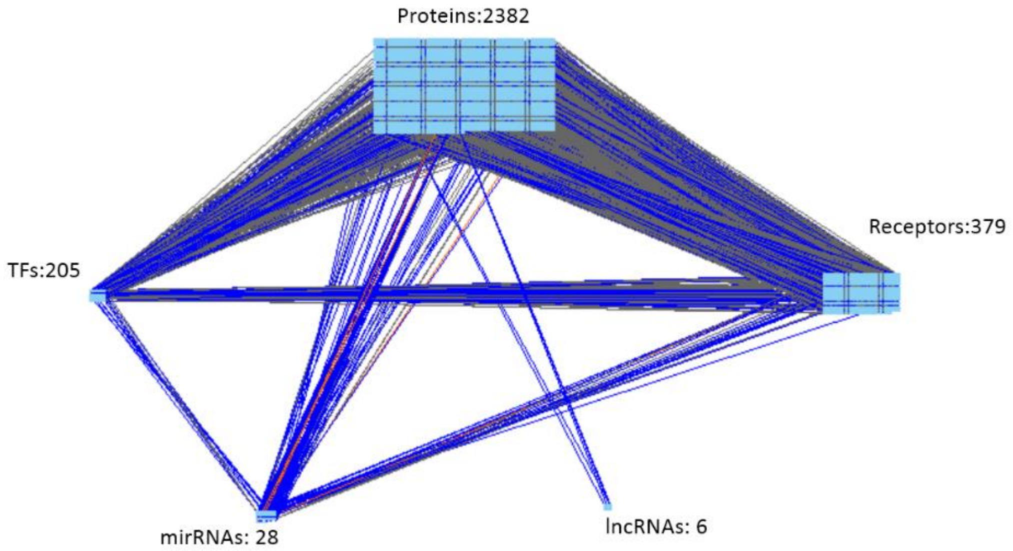

Figure S6. The core genome-wide genetic and epigenetic network (GWGEN) of normal prostate cells in the obese group. The numbers of Proteins, Receptors, TFs, miRNAs and lncRNA are 2382, 379, 205, 28 and 6, respectively.

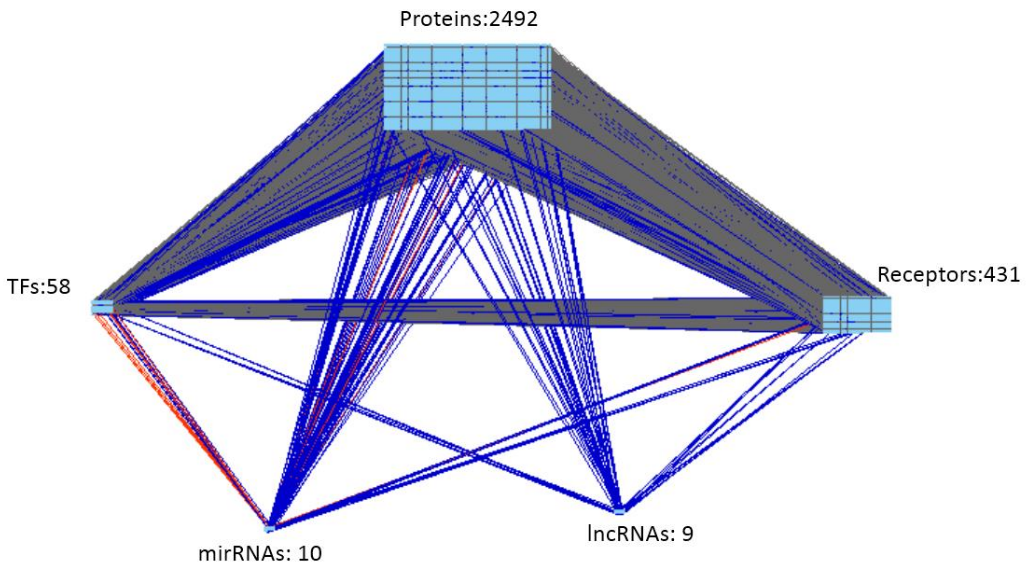

Figure S7. The core genome-wide genetic and epigenetic network (GWGEN) of lean PCa. The numbers of Proteins, Receptors, TFs, miRNAs and lncRNA are 2492, 431, 58, 10 and 9, respectively.

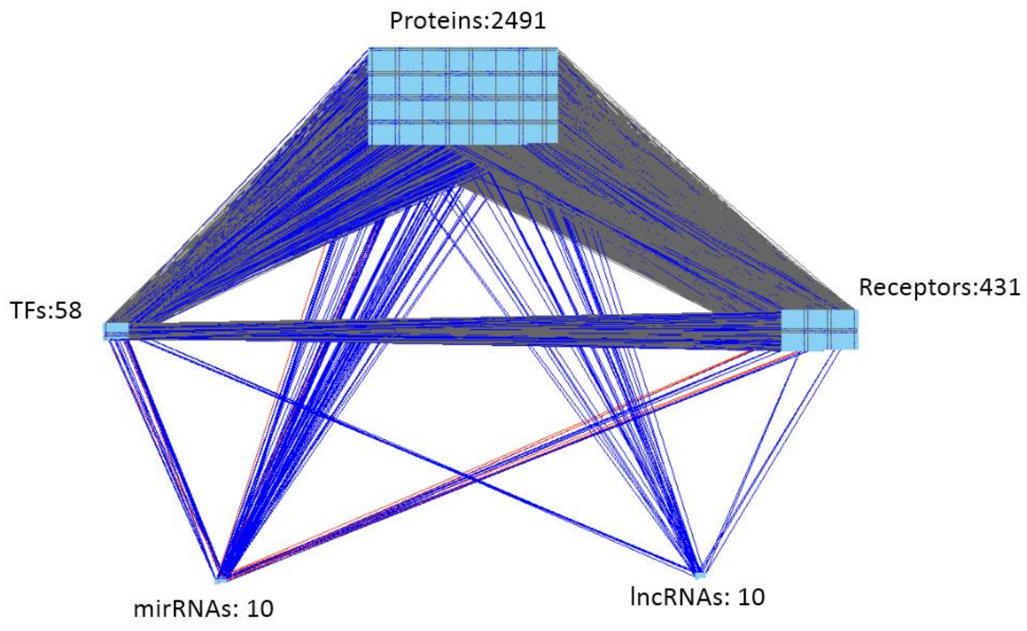

Figure S8. The core genome-wide genetic and epigenetic network (GWGEN) of obese PCa. The numbers of Proteins, Receptors, TFs, miRNAs and lncRNA are 2491, 431, 58, 10 and 10, respectively.

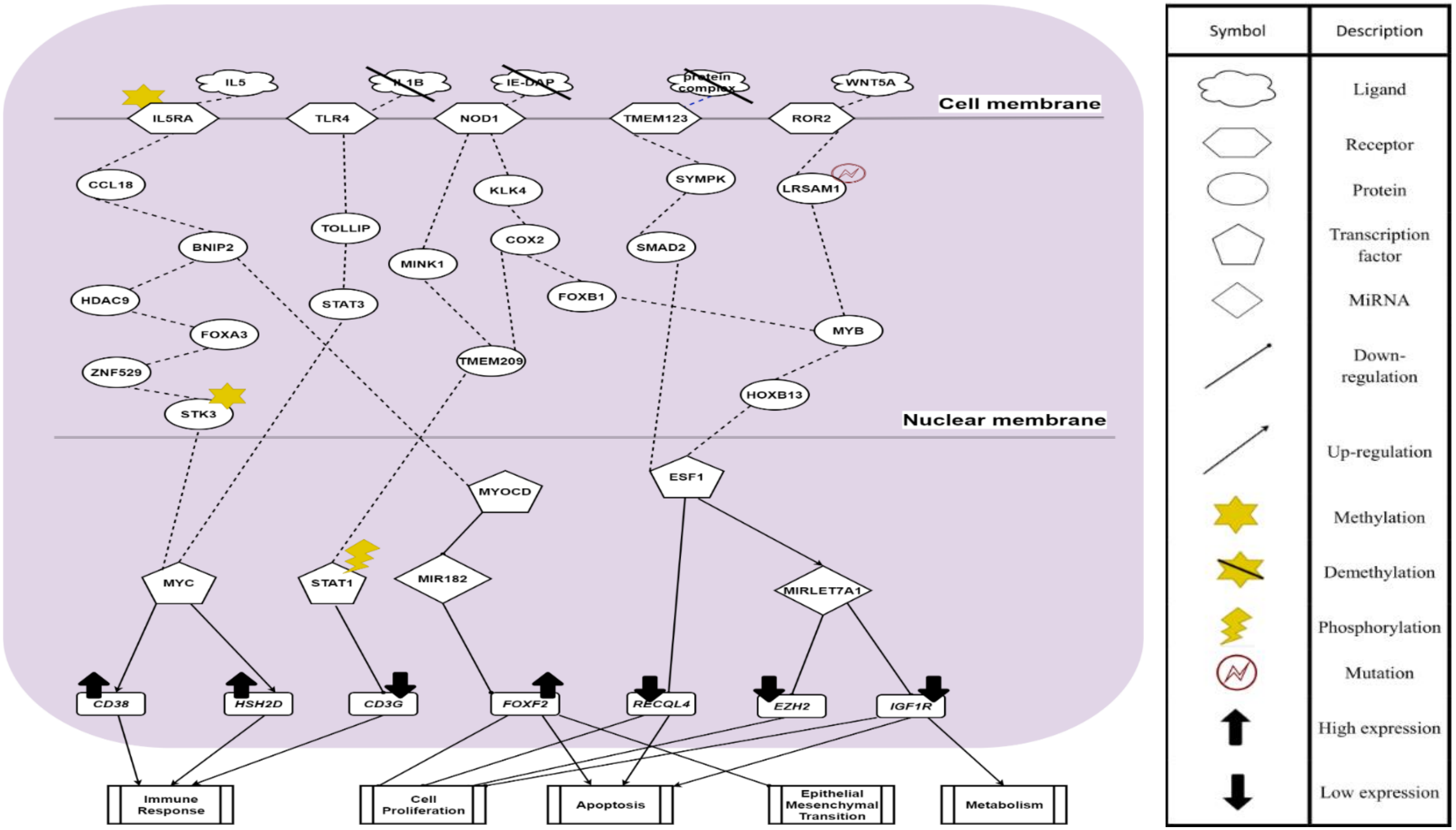

Figure S9. The core signaling pathways to investigate the healthy mechanism of normal prostate cells in the lean group. The light purple region indicates core signaling pathways of normal prostate cells in the lean group; the black arrow head of solid lines denotes activation of TF, miRNA, target genes and cellular functions; the black circle head of solid lines refers to inhibition of TF, miRNA, target genes and cellular functions; the black up arrow means high expression of target genes; the black down arrow indicates low expression of target genes.

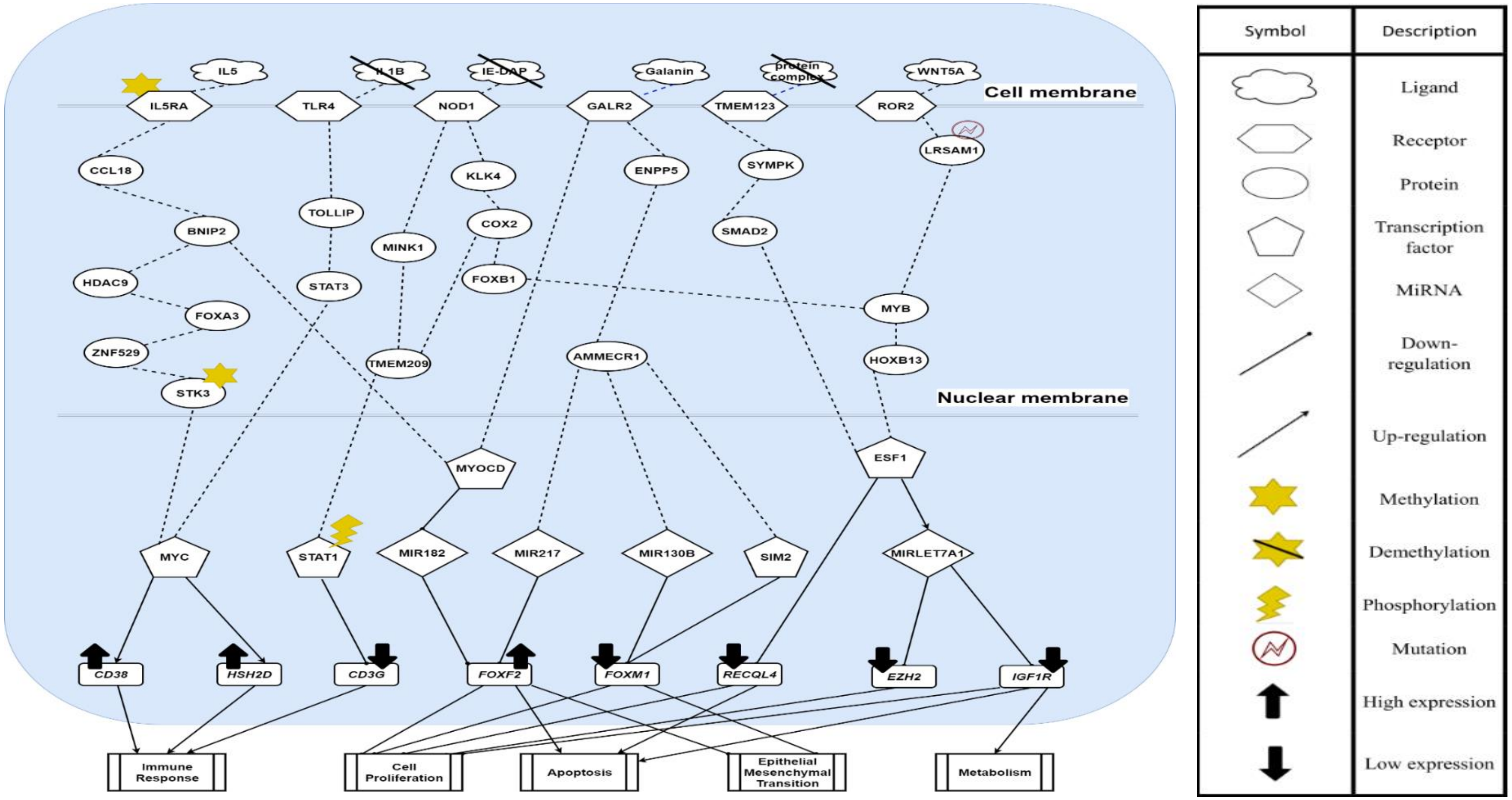

Figure S10. The core signaling pathways to investigate the healthy mechanism of normal prostate cells in the obese group. The light blue region indicates core signaling pathways of normal prostate cells in the obese group.

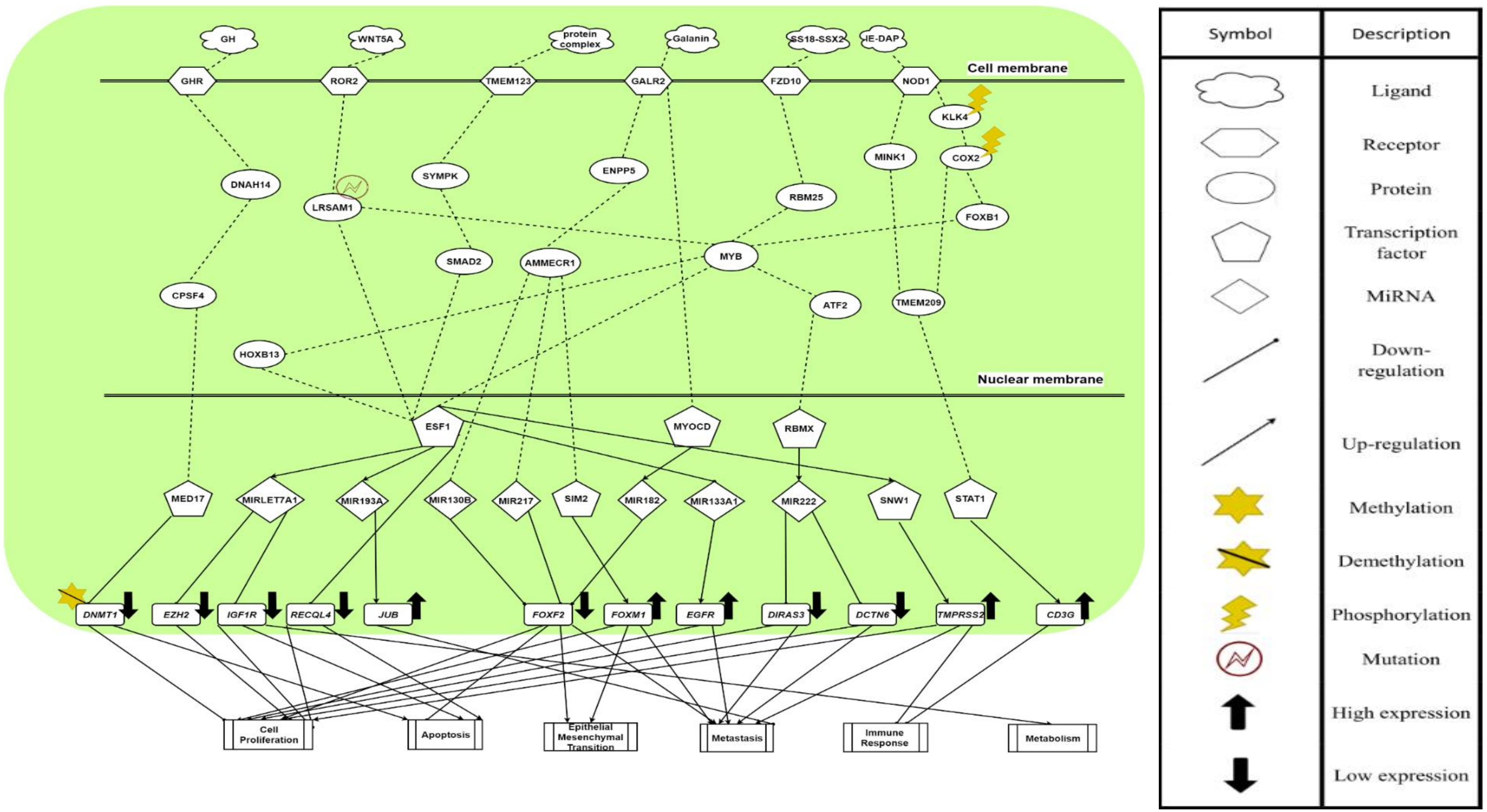

Figure S11. The core signaling pathways to investigate the carcinogenic mechanism of lean PCa. The light green region indicates core signaling pathways of lean prostate cancer.

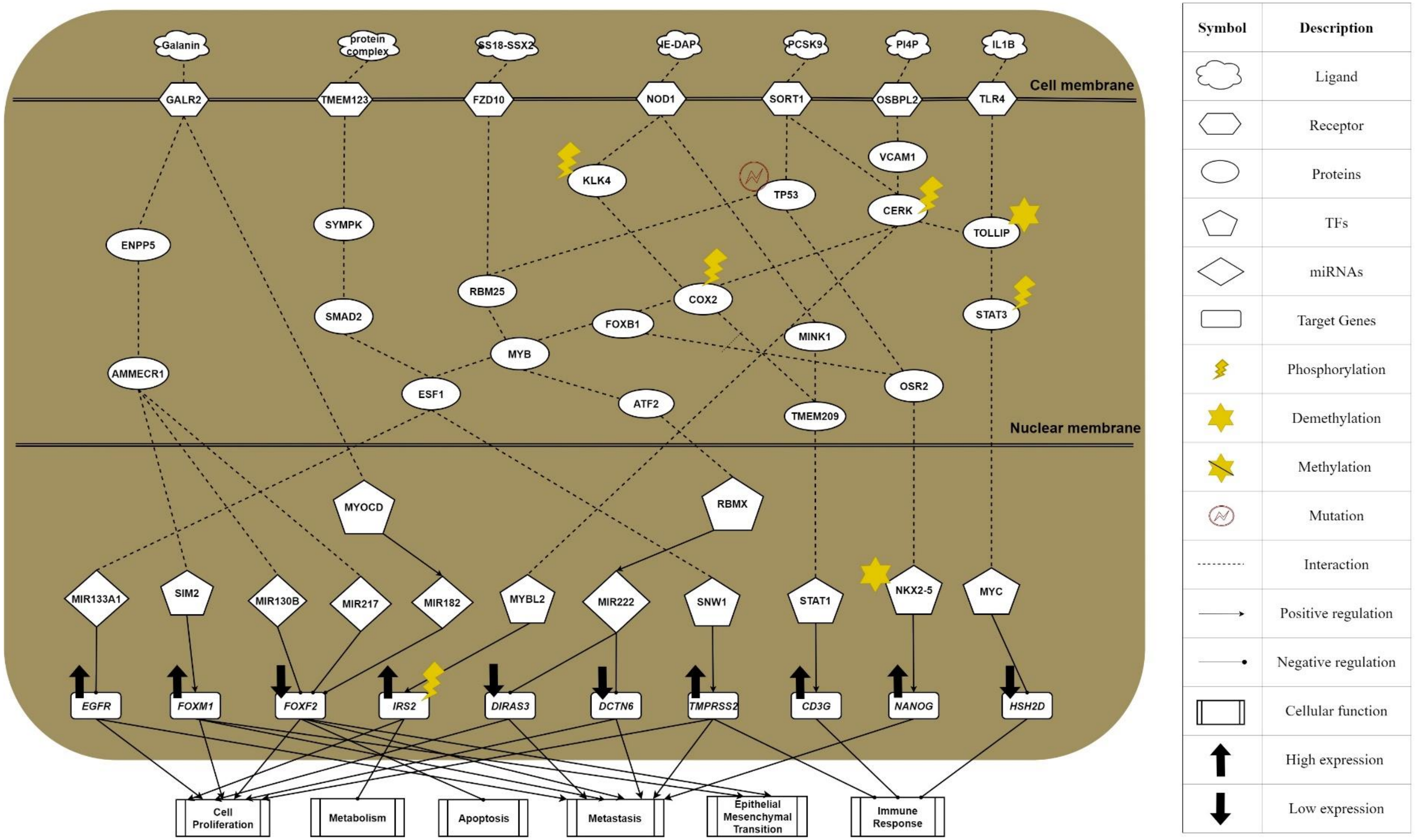

Figure S12. The core signaling pathways to investigate the carcinogenic mechanism of obese PCa. The brown region indicates core signaling pathways of obese prostate cancer.

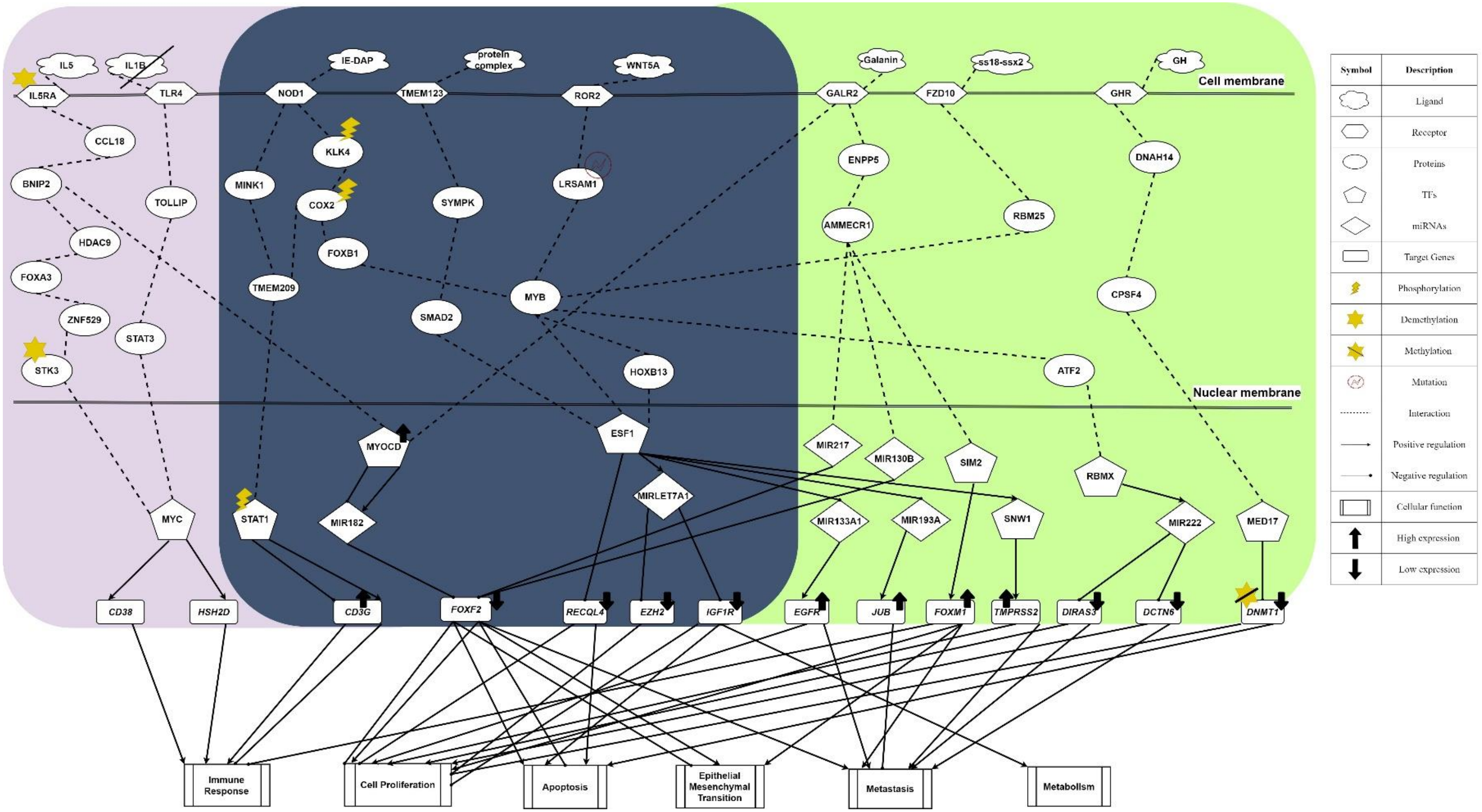

Figure S13. The core signaling pathways integrated from core signaling pathways of normal prostate cells (lean group) in Figure S9 and lean PCa in Figure S11. This figure summarizes the genetic and epigenetic carcinogenic mechanism of normal prostate cells in the lean group and lean PCa. The signaling pathways in the deep blue color region are the common core signaling pathways of normal prostate cells in the lean group and lean PCa; The light purple region represents specific core signaling pathways of normal prostate cells in the lean group; The light green region denotes specific core signaling pathways of lean PCa; the black arrow head of solid lines denotes activation of TF, miRNA, target genes and cellular functions; the black circle head of solid lines refers to inhibition of TF, miRNA, target genes and cellular functions; the black up arrow means high expression of protein, receptor, TF, and target genes; the black down arrow indicates low expression of protein, receptor, TF, and target genes.

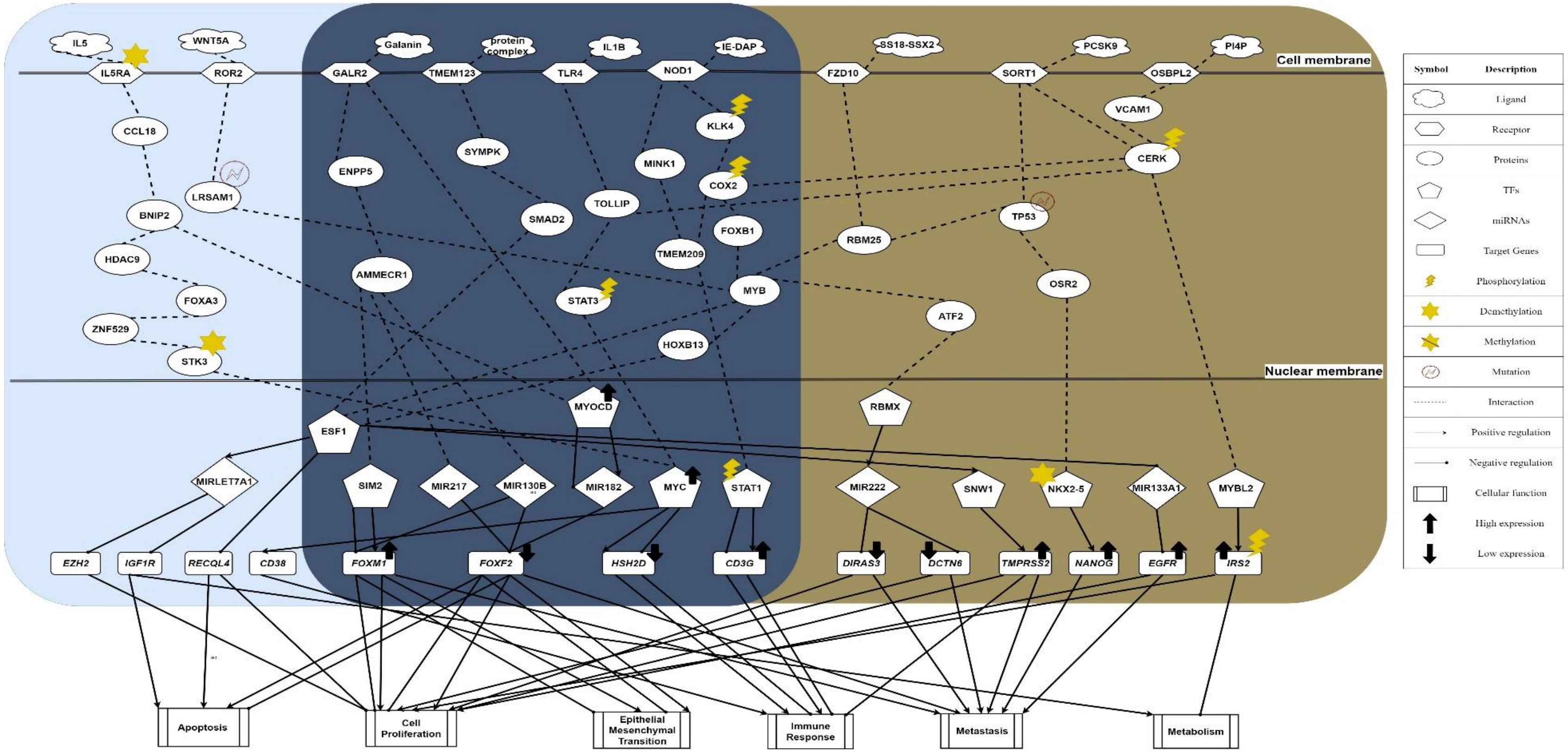

Figure S14. The core signaling pathways integrated from core signaling pathways of normal prostate cells (obese group) in Figure S10 and obese PCa in Figure S12. This figure summarizes the genetic and epigenetic carcinogenic mechanism of normal prostate cells in the obese group and obese PCa. The signaling pathways in the deep blue color region are the common core signaling pathways of normal prostate cells in the obese group and obese PCa; The light blue region represents specific core signaling pathways of normal prostate cells in the obese group; The brown color region denotes specific core signaling pathways of obese PCa; the black arrow head of solid lines denotes activation of TF, miRNA, target genes and cellular functions; the black circle head of solid lines refers to inhibition of TF, miRNA, target genes and cellular functions; the black up arrow means high expression of protein, receptor, TF, and target genes; the black down arrow indicates low expression of protein, receptor, TF, and target genes.

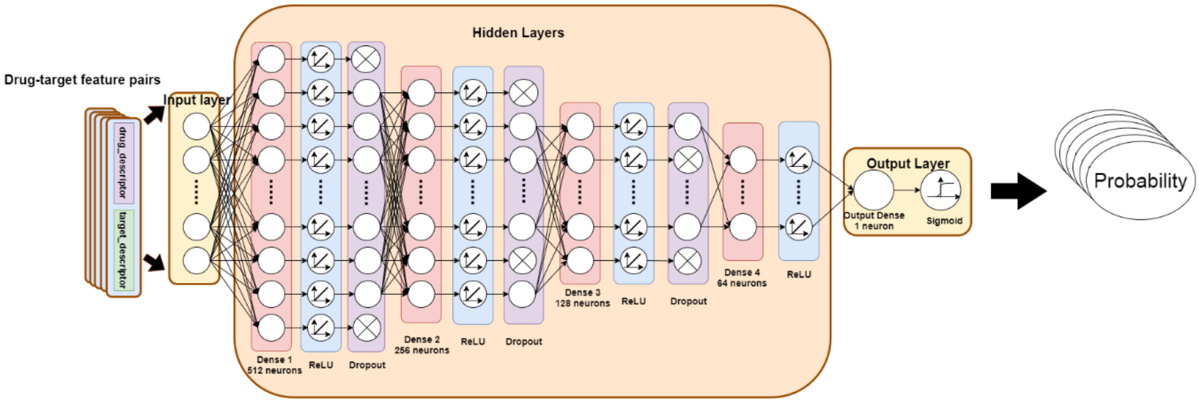

Figure S15. The structure of DTI model. In order to predict the docking of drug-target pairs, we constructed a neural network of four hidden layers and a ReLU activation function layer. Compared with other activation functions, ReLU activation function owns advantages of preventing gradients from disappearing and converging faster. Although ReLU is not good enough to cover every aspect of DNN field, it is effective for us to employ it on classification issues. For the purpose of avoiding overfitting, we merged the dropout layer into the rear process between ReLU and each hidden layer. Moreover, there are sequentially 512, 256, 128, and 64 neurons in four hidden layers and the input layer has a dimension of 694, related to the features of each drug and target. Next, after adopting a sigmoid activation function in the output layer to display properties of binary classification, we could limit the probability value to the scope of 0 and 1. Consequently, the outcome means that the higher the probability value is, the stronger the interaction of drug-target pairs is. Based on the reliable drug-target docking prediction in DTI model, the candidate drugs were picked out to target selected biomarkers in core signaling pathways of PCa.

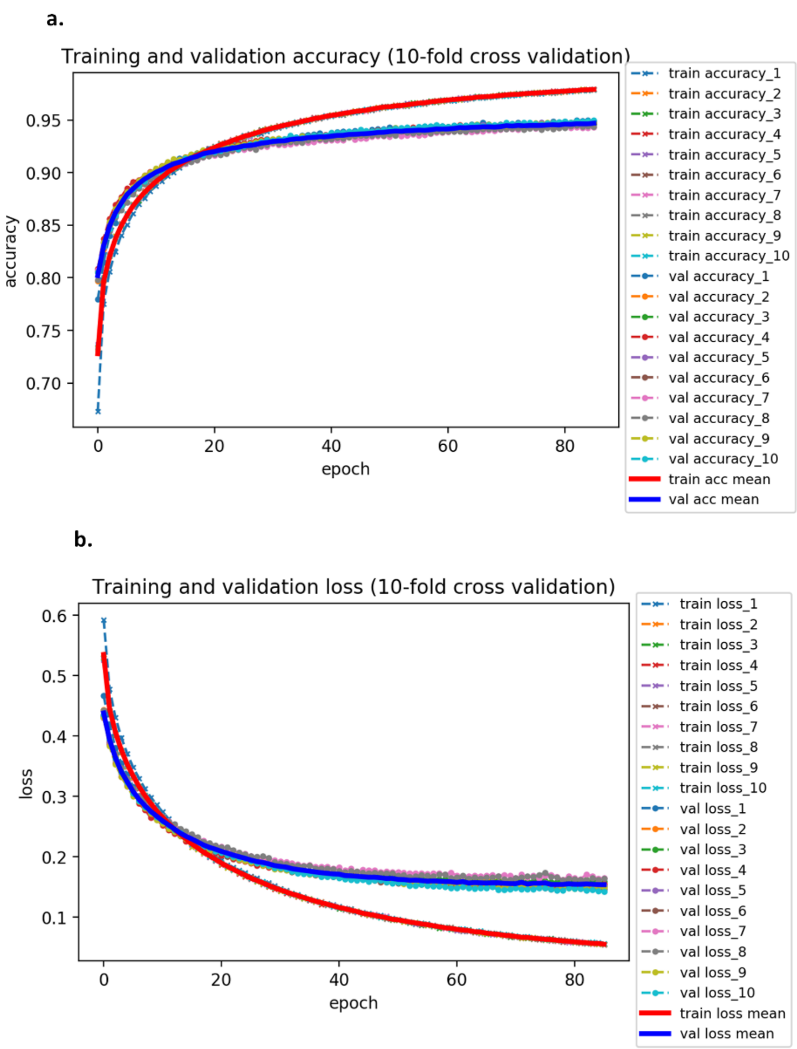

Figure S16. The accuracy and loss for training and validation sets by 10-fold cross validation. **a.** The training and validation accuracy by 10-fold cross validation. **b.** The training and validation loss by 10-fold cross validation.

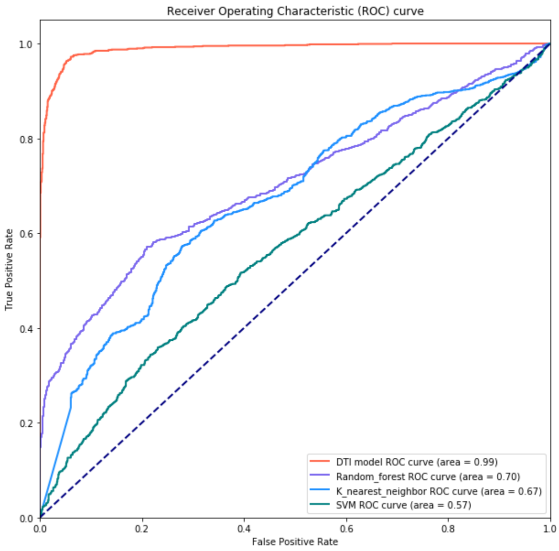

Figure S17. The ROC curves of different models for the drug-target interaction prediction. The dot line means the worst situation (AUC = 0.5) for models to make a distinction between positive and negative class.
